# Asymmetric Structure‐Driven Functional Synergy: A Multistimuli‐Activated Janus Nanomotor for Self‐Amplifying NO Cascade Therapy

**DOI:** 10.1002/advs.77848

**Published:** 2026-09-27

**Authors:** Chengcheng Li, Yu Zhang, Yu Chen, Lingyun He, Xian Shen, Weijian Sun, Hongbo Zhang

**Affiliations:** ^1^ Department of Colorectal and Anal Surgery Zhejiang–Finland Joint Laboratory of Gastrointestinal Tumor Metabolism and Nutrition The First Affiliated Hospital of Wenzhou Medical University Wenzhou Zhejiang China; ^2^ Pharmaceutical Sciences Laboratory Faculty of Science and Engineering Åbo Akademi University Turku Finland; ^3^ Turku Bioscience Centre University of Turku and Åbo Akademi University Turku Finland

**Keywords:** cancer therapy, gas therapy, nanomotor, NO, photothermal therapy

## Abstract

Nitric oxide (NO)‐based cancer therapy is fundamentally limited by poor intratumoral penetration of exogenous donors and their inability to sustain localized relevant flux. Here, a multistimuli‐activated Janus nanomotor was developed to interconnect nonenzymatic and enzymatic NO‐generation pathways, enabling in situ self‐cascading NO production. The nanomotor comprises an AuPt_3_Cu nanozyme asymmetrically coated with a disulfide‐bridged mesoporous silica shell (2sMSN) loaded with L‐arginine, allowing autonomous propulsion under O_2_, NO, and NIR irradiation to achieve deep tumor penetration. Upon cellular internalization, the asymmetric architecture enables concurrent AuPt_3_Cu‐catalyzed oxidation of glucose to H_2_O_2_ and GSH‐responsive degradation of 2sMSN to release L‐arginine, thereby coupling nonenzymatic NO generation through H_2_O_2_‐mediated L‐arginine oxidation. Subsequently, the resulting nitrosative stress suppresses glycolysis and drives compensatory pentose phosphate pathway activation, thereby increasing cofactor availability and enhancing nitric oxide synthase–dependent NO production. This self‐amplifying NO loop further amplifies mild photothermal therapy by inducing HSP90 downregulation, thereby triggering immunogenic cell death and achieving effective melanoma clearance. Overall, this Janus nanomotor presents an “asymmetric structure‐driven functional synergy” strategy for controllable, sustained, and uniform NO generation, offering a distinctive paradigm to overcome the intrinsic penetration and sustainability limitations of gas‐based therapeutic modalities.

## Graphical abstract

The Janus nanomotors leverage an “asymmetric structure‐driven functional synergy” strategy to orchestrate spatiotemporally coordinated catalytic functions, in situ self‐amplifying NO generation, and deep tumor penetration, thereby enabling controllable, sustained, and uniform NO generation within the tumor microenvironment.

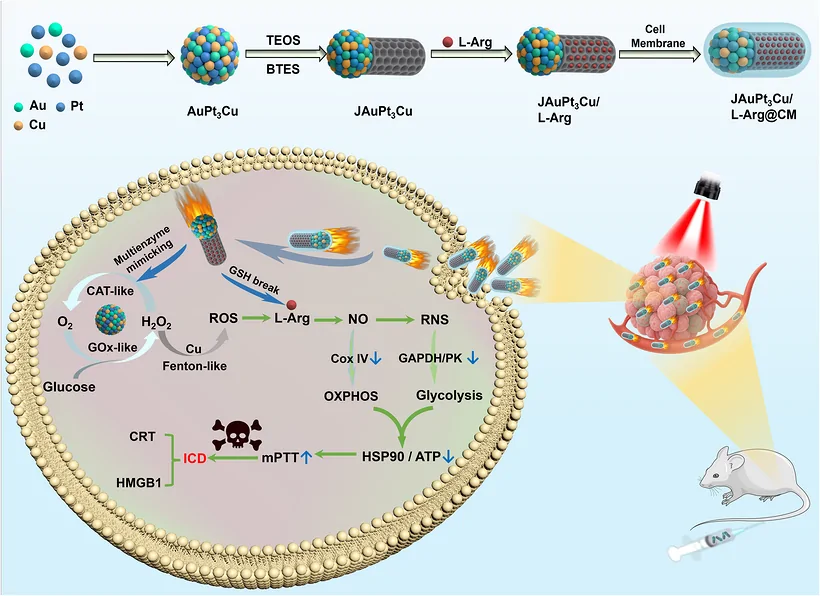

## 1 Introduction

Gas therapy has emerged as a novel therapeutic strategy that utilizes bioactive gaseous molecules to regulate key biological processes in cancer treatment. Among these, nitric oxide (NO)‐based gas therapy has attracted significant attention due to its unique chemical reactivity and broad regulatory roles in oncology [1, 2]. However, the biological effects of NO in tumors are highly dependent on its localized flux. Transient, low levels of NO can promote angiogenesis and impair antitumor immune surveillance, favoring tumor progression, whereas sustained, high concentrations of NO disrupt redox homeostasis and suppress cellular energy metabolism, triggering tumor cell death [3, 4, 5]. Therefore, sustaining high local NO flux within the tumor microenvironment (TME) is a fundamental requirement for NO‐based cancer therapy.

Conventional exogenous NO donors, including nitrates, nitrites, and various small‐molecule prodrugs, suffer from inherent limitations such as nonspecific bioactivation, potential systemic toxicity, and insufficient tissue penetration [6, 7, 8]. In contrast, the endogenous substrate L‐arginine‐based in situ NO generation strategy offers superior controllability and biocompatibility, but both the nonenzymatic and enzymatic pathways mediating this process are constrained by the complexity of the TME [9, 10].

In the nonenzymatic pathway, L‐arginine can undergo chemical oxidation by hydrogen peroxide (H_2_O_2_) and other reactive oxygen species (ROS) to generate NO [11, 12]. However, the spatial heterogeneity and temporal dynamics of ROS levels within the TME result in discrete reaction sites and fluctuating NO levels that fail to sustain therapeutic concentrations of NO [13, 14]. In the enzymatic pathway, NO production relies on nitric oxide synthase (NOS)‐catalyzed oxidation of L‐arginine, which requires oxygen, NADPH, and other cofactors to support continuous catalysis. Even in melanoma, where inducible NOS is upregulated, NO production remains limited by insufficient substrate availability and local hypoxia [15, 16, 17]. More critically, conventional nanocarriers predominantly accumulate around perivascular regions and tumor margins instead of penetrating deep into tumor tissue [18, 19]. This limited spatial delivery amplifies the spatiotemporal heterogeneity of NO production, preventing the establishment of a continuous, therapeutically relevant NO flux across the entire tumor mass. These challenges fundamentally limit the antitumor efficacy of NO‐based gas therapy.

**SCHEME 1:**
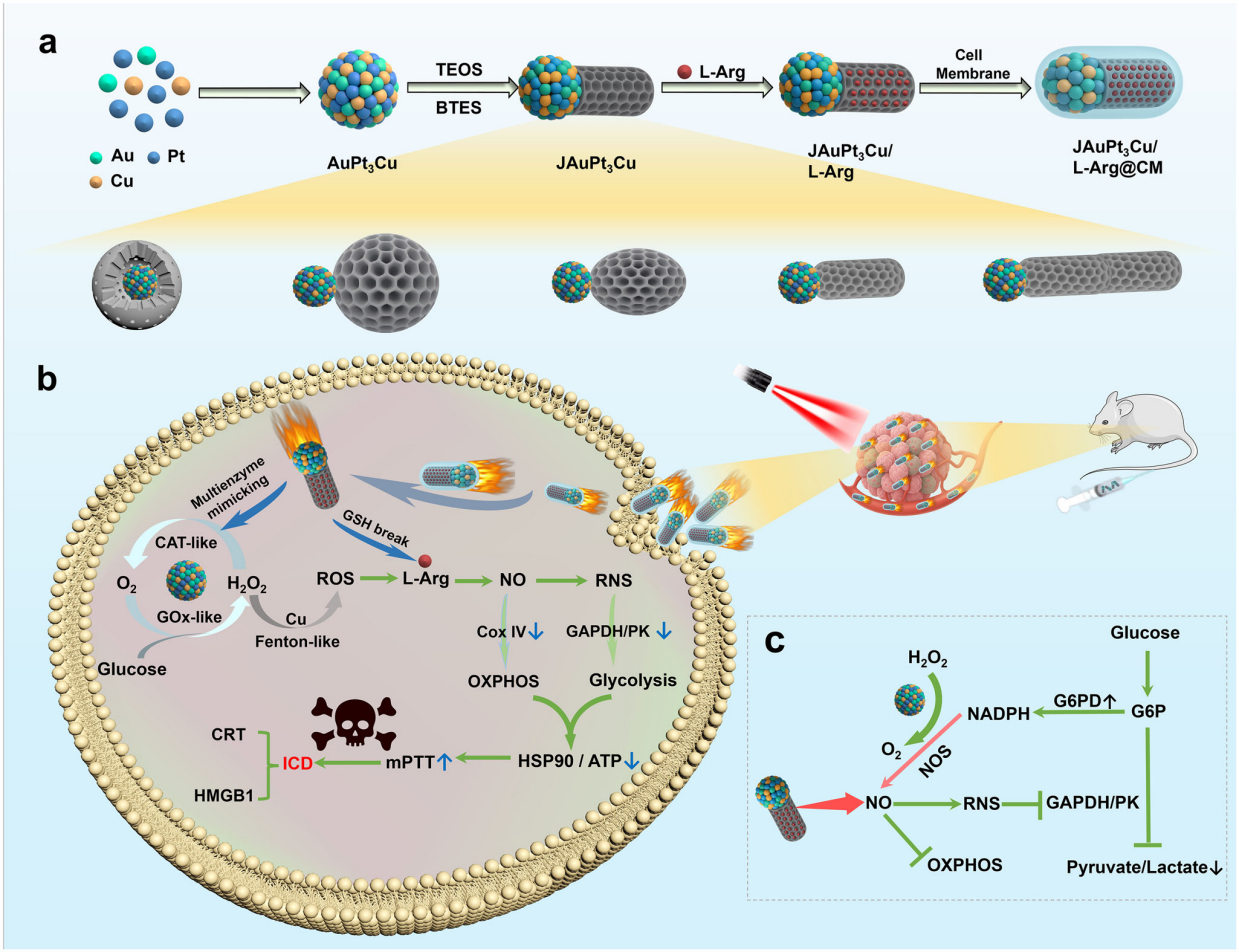
Preparation and antitumor mechanism of JAPC/A@CM. (a) Schematic illustration of the preparation process for Janus nanomotor JAPC/A@CM. (b) Mechanistic elucidation of NO‐driven multimodal therapeutics mediated by JAPC/A@CM. (c) Mechanistic schematic of in situ self‐cascading NO production.

Herein, to address the spatiotemporal heterogeneity of intratumoral NO generation, we introduce a multistimuli‐activated Janus nanomotor based on an “asymmetric structure‐driven functional synergy” strategy, enabling controllable, sustained, and uniform NO generation within the tumor microenvironment. This nanomotor (JAPC/A@CM) consists of an AuPt_3_Cu nanozyme (glucose oxidase‐like (GOx‐like), peroxidase‐like (POD‐like), and catalase‐like (CAT‐like) activities) asymmetrically coated with a glutathione (GSH)‐responsive disulfide‐bridged mesoporous silica shell loaded with L‐arginine (Scheme 1a).

This asymmetric Janus architecture enables AuPt_3_Cu‐mediated catalysis to drive an H_2_O_2_/O_2_/ROS product cycle, while the GSH‐responsive 2sMSN shell continuously releases L‐arginine within the TME, thereby coupling the nonenzymatic oxidation pathway (Scheme 1b). The resulting NO can induce nitrosative stress and drive metabolic reprogramming to increase NADPH production. The accumulated NADPH, together with O_2_ generated by the AuPt_3_Cu nanozyme, further enhances enzymatic NO‐generation pathways (Scheme 1c). More importantly, the nanomotor can actively penetrate deep tumor regions under multiple endogenous (H_2_O_2_ and L‐arginine) and exogenous cues (NIR irradiation), thereby overcoming perivascular accumulation and achieving uniform intratumoral distribution of NO. Moreover, this self‐amplifying NO cascade can further enhance mild photothermal therapy, thereby supporting effective melanoma treatment. Collectively, this Janus nanomotor establishes an “asymmetric structure‐driven functional synergy” design concept that leverages active intratumoral transport, metabolic feedback, and dual‐pathway coupling to overcome the intrinsic limitations of conventional NO delivery, offering a novel paradigm for gas‐based cancer therapies.

## 2 Results

### 2.1 Synthesis and Characterization of JAPC/A@CM

**FIGURE 1:**
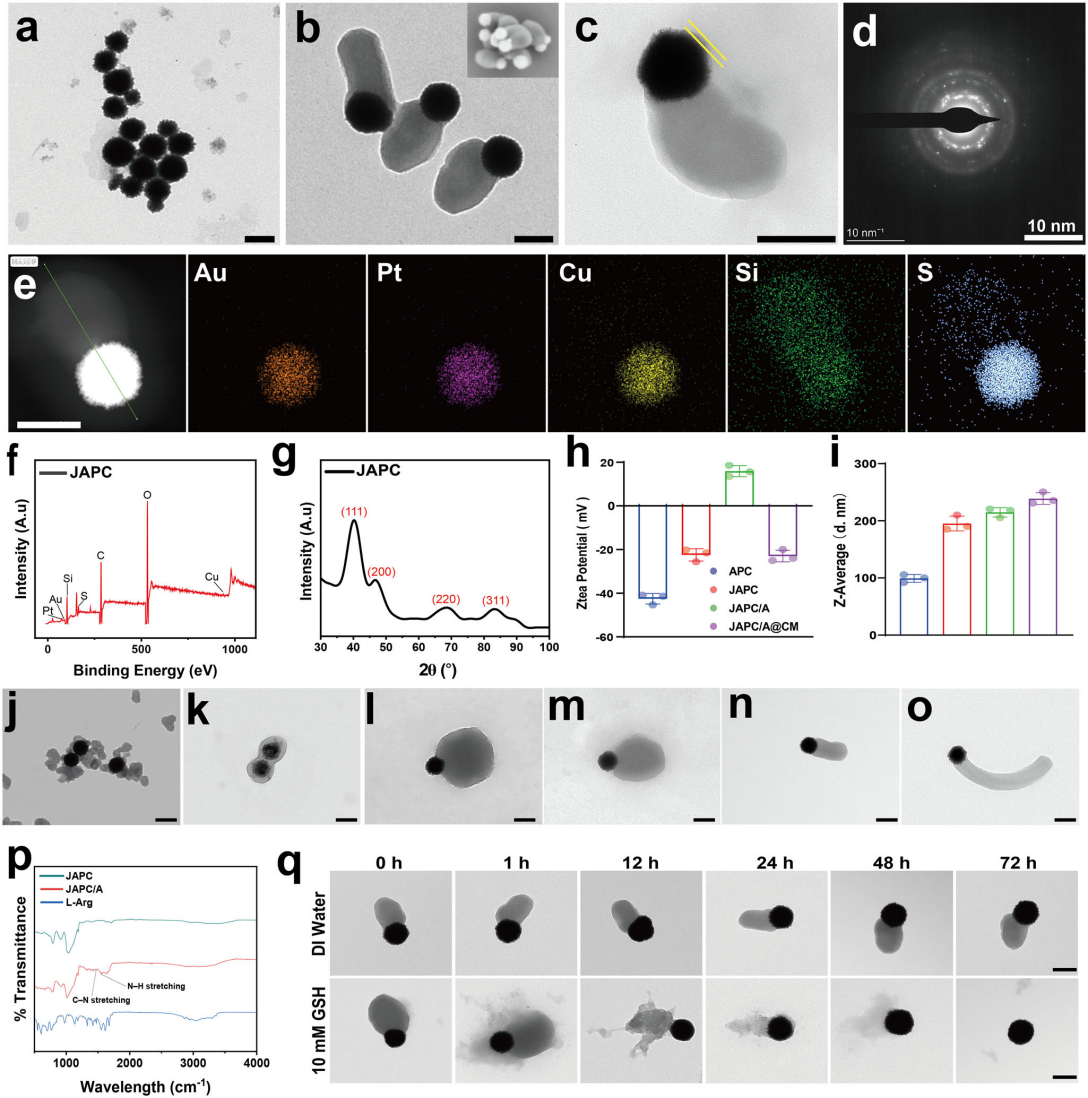
Characterization of Janus nanomotor JAPC/A@CM. (a) TEM images of AuPt_3_Cu, (b) JAPC, (c) JAPC/A@CM. (d) SAED of JAPC. (e) Elemental mapping images of JAPC. (f) XPS spectra of JAPC. (g) XRD pattern of JAPC. (h) Zeta potential of various nanoparticles. (i) Hydrodynamic diameter of various nanoparticles. TEM images of nanoparticles synthesized under different conditions: (j) high ammonia concentration, 0.8 m; (k) low ammonia concentration, 0.15 m; (l) ethanol concentration, 3.0 m; (m) ethanol concentration, 2.0 m; (n) ethanol concentration, 1.0 m; and (o) silica precursor concentration, 5 mm. (p) FTIR spectrum of various nanoparticles. q) TEM images for GSH‐responsive degradation of JAPC/A@CM. All scale bars: 80 nm.

The synthetic procedure of JAPC/A@CM is illustrated in Scheme 1a. AuPt_3_Cu nanozymes were first prepared through a one‐pot reduction approach based on a previously reported method with slight modifications [20]. Briefly, PtCl_6_^2^^−^, AuCl_4_^−^, and Cu^2^^+^ ions were co‐reduced by L‐ascorbic acid at room temperature. As shown in Figure 1a, the resulting AuPt_3_Cu nanoparticles possessed a rough‐surfaced spherical morphology. Dynamic light scattering (DLS) measurements indicated a hydrodynamic diameter of 99.2 nm and a zeta potential of −42.5 mV (Figure 1h,i).

Subsequently, the AuPt_3_Cu nanoparticles were asymmetrically coated with disulfide‐bond‐bridged mesoporous silica through a sol–gel process to form the Janus architecture (JAPC). As shown in Figure 1b and Figure S1, the obtained particles exhibited a characteristic ball‐and‐rod morphology, in which an elongated mesoporous silica segment was attached to the metallic spherical core. Elemental mapping analysis (Figure 1e) revealed that Au, Pt, and Cu elements were mainly concentrated in the spherical head, whereas Si and S elements were distributed throughout the rod‐like mesoporous silica region, confirming the semi‐encapsulation structure. Due to background interference and peak overlap between Si/S signals and the M‐lines of Au/Pt, Si and S signals also appeared in the alloy core region. EDS spectra (Figure S2) further verified the elemental composition and spatial distribution within JAPC, supporting the formation of the Janus heterostructure. Additionally, x‐ray photoelectron spectroscopy (XPS) results (Figure 1f and Figure S3) confirmed the presence of Au, Pt, Cu, S, and Si species in JAPC nanoparticles, suggesting the successful synthesis of JAPC.

Selected‐area electron diffraction (SAED) patterns of JAPC (Figure 1d) showed distinct diffraction rings, demonstrating the polycrystalline nature of the AuPt_3_Cu domain and indicating that the asymmetric silica growth did not disturb the crystalline framework of the metal core. This was further corroborated by x‐ray diffraction (XRD) analysis (Figure 1g), where JAPC displayed characteristic reflections corresponding to the (111), (200), (220), and (311) planes of the face‐centered cubic AuPt_3_Cu phase. Together, these results confirm that asymmetric mesoporous silica coating preserves the intrinsic crystallinity of the alloy core.

In addition, the influence of synthetic parameters on the formation of the Janus nanoparticles was further explored. As shown in Figure 1j, at high ammonia concentrations (0.8 m), homogeneous nucleation was dominant, leading to the generation of free mesoporous silica nanoparticles without deposition on the alloy cores. When the ammonia level was reduced to 0.15 m, heterogeneous nucleation became favored, producing core–shell structures (Figure 1k). Under a fixed CTAB concentration, the ethanol content exerted a pronounced influence on the morphology of mesoporous silica. As shown in Figure 1l–n, decreasing the ethanol fraction (3.0, 2.0, and 1.0 m) led to increasingly anisotropic growth of the silica domains, resulting in a gradual transition from spherical to rod‐like morphologies. This behavior can be attributed to ethanol‐dependent modulation of interfacial energy and CTAB micellization, where reduced ethanol content enhances hydrophobic interactions and headgroup electrostatic repulsion, favoring the formation of elongated micellar templates that direct anisotropic silica deposition. Moreover, increasing the silica precursor (5 mm) concentration promoted directional growth of the mesoporous silica, resulting in more elongated MSN rods (Figure 1o). Collectively, these results demonstrate that precise regulation of the synthetic parameters enables controlled anisotropy and architecture of Janus nanoparticles, providing a rational strategy to optimize both the propulsion behavior and cargo‐loading capacity of the nanomotors.

L‐arginine was then loaded into the mesoporous silica framework of JAPC (JAPC/A). Successful L‐arginine loading was confirmed by an increase in zeta potential from −22.4 to +15.9 mV, along with characteristic guanidinium group peaks in the Fourier Transform Infrared (FTIR) spectrum, corresponding to C─N and N─H stretching vibrations (Figure 1p). To confer homologous tumor‐targeting capability, a cancer cell membrane derived from A375 cells was subsequently assembled onto the JAPC/A particles (JAPC/A@CM) following a previously reported protocol [21]. TEM imaging (Figure 1c) revealed a membrane layer of approximately 3.5 nm in thickness, and the accompanying decrease in zeta potential to around −23.0 mV further confirmed successful cell membrane adsorption.

The structural stability and GSH‐responsive behavior of JAPC/A@CM were evaluated by incubating the nanoparticles in DI water or DI water containing 10 mm GSH at pH 6.5 for 72 h. TEM results (Figure 1q) showed that the Janus structure remained intact in DI water, whereas the 2sMSN shell of JAPC/A@CM gradually degraded in the presence of GSH. These findings collectively confirm the successful construction of JAPC/A@CM and its responsiveness to intracellular GSH levels.

### 2.2 Asymmetric Structure–Driven Nonenzymatic NO Generation

L‐arginine can react nonenzymatically with H_2_O_2_ or other ROS to produce NO [11]. In this Janus system, the partially coated 2sMSN shell preserves the GOx‐, CAT‐, and POD‐like catalytic activities of the AuPt_3_Cu core [20], enabling continuous ROS generation while simultaneously allowing GSH‐responsive release of L‐arginine. This “asymmetric structure‐driven functional synergy” design supports a stepwise catalytic process that enables spatiotemporally controlled NO generation within the tumor microenvironment. The generated NO can further react with ROS to form reactive nitrogen species (RNS), thereby amplifying oxidative and nitrosative stress [22]. To verify this mechanism, we first evaluated the in vitro ROS‐producing capacity of JAPC/A through its triple enzyme‐like cascades.

**FIGURE 2:**
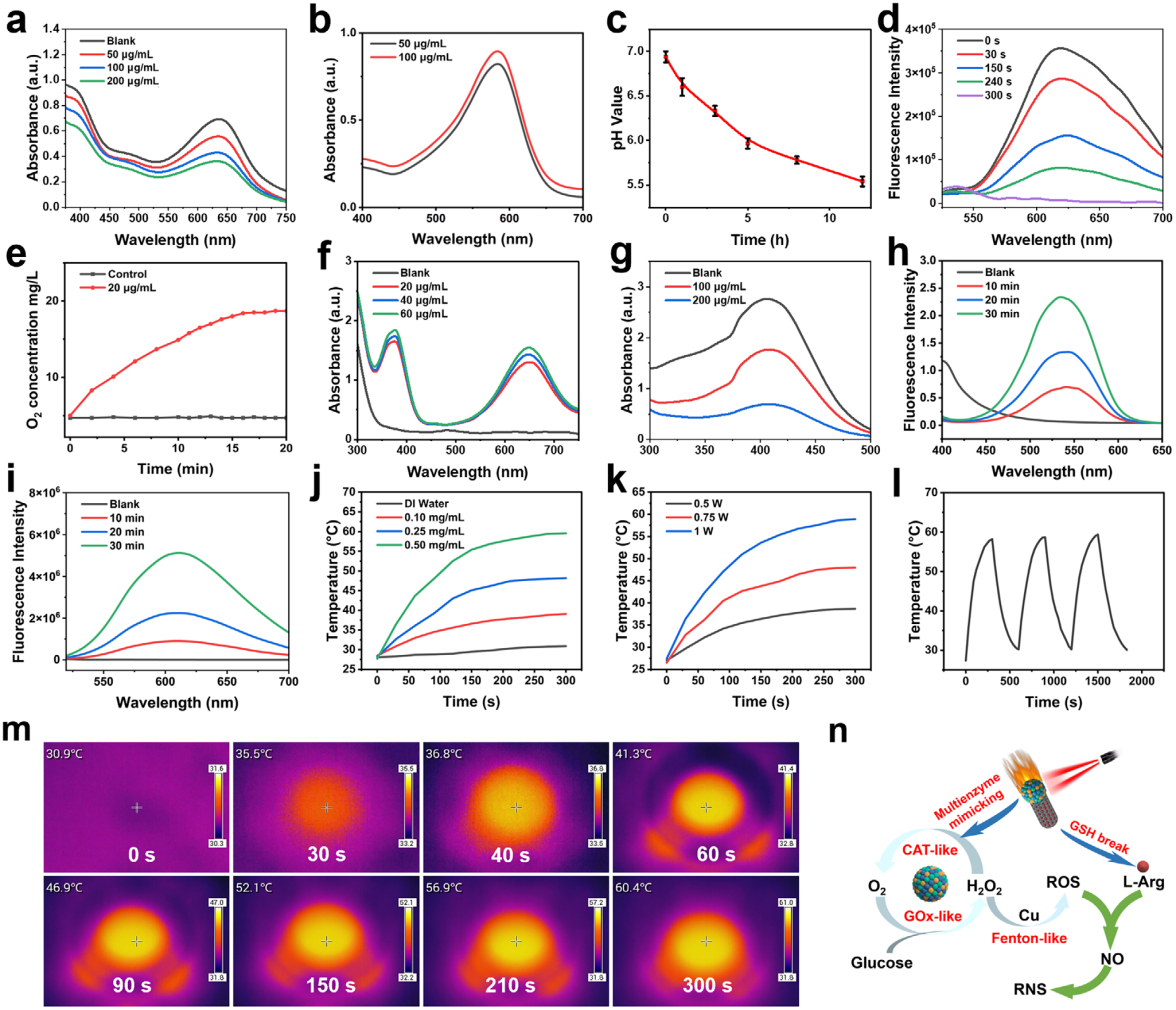
In vitro evaluation of NO/RNS cascade generation and photothermal conversion mediated by JAPC/A@CM. (a) Glucose degradation and (b) H_2_O_2_ generation capacity of JAPC/A@CM at different concentrations. (c) pH variation during the incubation of JAPC/A@CM with glucose solution. (d) Oxygen production detected using a hypoxia‐sensitive fluorescence probe. (e) Quantitative O_2_ generation profiles of JAPC/A@CM at various concentrations. (f) POD‐like activity evaluation using TMB assay. (g) GSH depletion capacity of JAPC/A@CM. (h) Nonenzymatic NO generation mediated by JAPC/A@CM. (i) ONOO^−^ fluorescent probe detection using Rhodamine B hydrazide. (j) Photothermal performance of JAPC/A@CM at different concentrations under 808 nm laser irradiation. (k) Photothermal heating curves of JAPC/A@CM under different 808 nm laser powers. (l) Photothermal stability over three on/off cycles. (m) Infrared thermal imaging of JAPC/A@CM under 808 nm irradiation. (n) Schematic illustration of NO/RNS cascade generation mediated by the enzyme‐mimicking activity of JAPC/A@CM.

The GOx‐like activity of JAPC/A@CM was first assessed by monitoring glucose depletion, H_2_O_2_ generation, and gluconic acid formation. As shown in Figure 2a, as the concentration of AuPt_3_Cu increased, glucose consumption rose accordingly, accompanied by higher H_2_O_2_ levels (Figure 2b). A gradual decrease in the solution pH was also detected as the incubation time increased (Figure 2c). These results indicate efficient oxidation of glucose to gluconic acid, confirming the GOx‐like activity of AuPt_3_Cu. The generated H_2_O_2_ subsequently served as a substrate for the CAT‐ and POD‐like reactions [23]. The CAT‐like activity was subsequently evaluated by monitoring O_2_ generation during H_2_O_2_ decomposition. As shown in Figure 2d, a time‐dependent decrease in the fluorescence intensity of Ru(dpp)_3_Cl_2_ at 613 nm was observed, indicating continuous O_2_ production. This result was further corroborated by dissolved oxygen measurements (Figure 2e), which demonstrated that higher concentrations of AuPt_3_Cu led to increased O_2_ generation. In addition, the POD‐like activity of JAPC/A was assessed using 3,3′,5,5′‐tetramethylbenzidine (TMB) oxidation. As shown in Figure 2f, increasing concentrations of JAPC/A@CM resulted in progressively enhanced ROS production, as evidenced by the intensified blue coloration of oxidized TMB (Figure S4). Together, these results show that JAPC/A@CM retains the Janus structural functionality while exhibiting effective tri‐enzyme–like catalytic behavior, providing steady ROS generation to support subsequent nonenzymatic NO formation.

The ability of AuPt_3_Cu to catalyze the reaction between L‐arginine and H_2_O_2_ for NO generation was next evaluated. As shown in Figure 2g, JAPC/A@CM efficiently consumed GSH, indicating GSH‐triggered cleavage of the disulfide bonds within the 2sMSN shell. This observation was consistent with the structural degradation of the mesoporous silica shell observed in the TEM images (Figure 1q), confirming the GSH‐responsive release of L‐arginine. Because GSH interferes with NO detection, nonenzymatic NO generation was directly assessed using an L‐arginine + JAPC/A@CM + H_2_O_2_ system. As shown in Figure 2h, NO levels increased progressively over time, confirming effective NO production mediated by the catalytic AuPt_3_Cu core of JAPC/A@CM.

More importantly, it was found that Cu^2^^+^ doping was essential for this process. As shown in Figure S5, the AuPt_3_Cu nanozyme generated noticeably more gas bubbles than AuPt_3_ under the same conditions. Further experiments (Figure S6) showed that L‐arginine and H_2_O_2_ alone produced only a small number of bubbles, whereas the addition of Cu^2^^+^ led to pronounced bubble formation. In contrast, Cu^2^^+^ incubated solely with either H_2_O_2_ or L‐arginine did not generate any NO bubbles. These observations suggest that Cu^2^^+^ may promote a Fenton‐type reaction that accelerates ROS production and thereby enhances the NO‐forming process [22, 24, 25]. Moreover, AuPt_3_Cu generates O_2_ via its CAT‐like activity, thereby facilitating the ROS‐mediated conversion of NO to RNS [26, 27, 28]. This was supported by peroxynitrite (ONOO^−^) probe measurements (Figure 2i), which revealed a time‐dependent increase in RNS levels concurrent with NO generation, indicating the conversion of NO into RNS. Overall, these results confirm that the asymmetric architecture enables AuPt_3_Cu‐mediated catalysis to drive an H_2_O_2_/O_2_/ROS product cycle, while the GSH‐responsive 2sMSN shell continuously releases L‐arginine, thereby establishing a multistep cascade that sustains NO and RNS production.

### 2.3 Photothermal Conversion Performance of JAPC/A

Au‐based nanomaterials exhibit strong photothermal conversion capability under NIR irradiation [29]. Given the potential synergy between NO signaling and photothermal effects, the photothermal behavior of JAPC/A@CM was evaluated under 808 nm laser irradiation. As shown in Figure 2j, JAPC/A@CM suspensions at concentrations of 0.10, 0.25, and 0.5 mg·mL^−^^1^ exhibited temperature increases after 5 min of laser irradiation, with higher concentrations resulting in greater temperature elevations. At 0.5 mg·mL^−^^1^, the temperature rise reached 31.9°C, whereas DI water displayed only a 2.8°C increase under the same conditions. In addition, the photothermal heating behavior was strongly dependent on the laser power (Figure 2k). Notably, the photothermal performance remained stable over three consecutive laser on/off cycles, demonstrating its excellent photothermal stability (Figure 2l). Infrared thermal imaging further confirmed the photothermal capability of JAPC/A@CM, with the maximum temperature of JAPC/A suspensions reaching 60.4°C within 5 min of laser irradiation at 1 W cm^−^^2^ (Figure 2m). These results indicate that JAPC/A@CM maintains reliable and controllable photothermal performance, supporting its potential in photothermal therapy and NIR‐guided nanomotor applications.

Collectively, as illustrated in Figure 2n, the nanozyme component of JAPC/A@CM exhibits integrated GOx‐, CAT‐, and POD‐like enzyme activities, enabling ROS generation through a cascade pathway. Following GSH‐triggered release of L‐arginine from the MSN component, ROS acts as a key mediator to enhance NO production from L‐arginine and subsequently drives the conversion of NO into RNS. Together with its photothermal capability, JAPC/A@CM provides a multifunctional platform that couples enzyme‐mimicking catalysis, gas‐mediated signaling, and NIR‐responsive heating, offering broad potential for combined cancer therapy.

### 2.4 Biocompatibility of JAPC/A@CM

**FIGURE 3:**
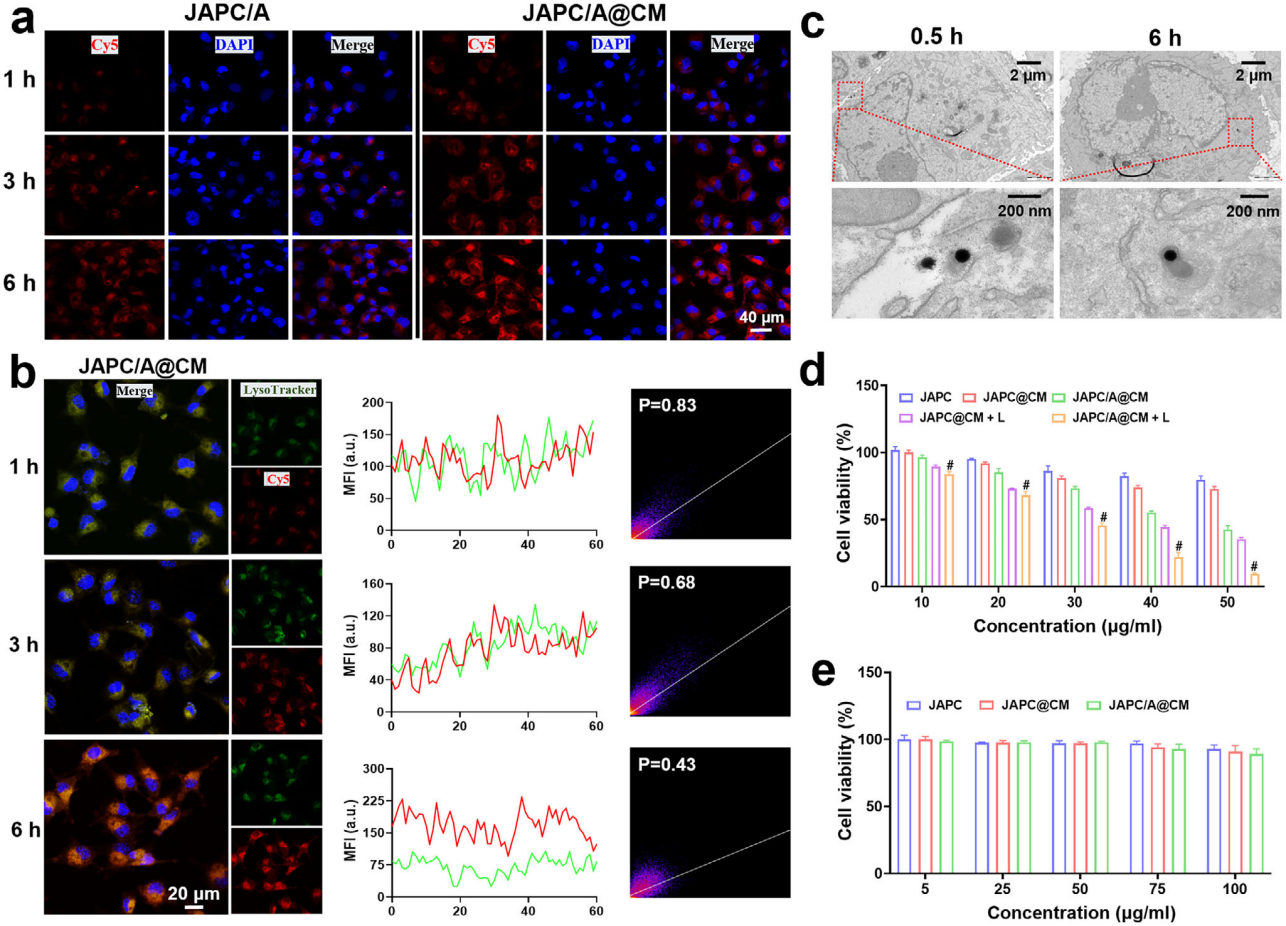
The biocompatibility of JAPC/A@CM. (a) CLSM images of A375 cells incubated with JAPC/A and JAPC/A@CM for 1, 3, and 6 h. (b) Colocalization of JAPC/A@CM with lysosomes in A375 cells after incubation for 1, 3, and 6 h. (c) Bio‐TEM images of A375 cells incubated with JAPC/A@CM for 0.5 and 6 h. (d) The viability of A375 cells and (e) HUVEC after various treatments. Statistical analysis was performed using one‐way analysis of variance (ANOVA) followed by Tukey's multiple‐comparisons test. All data are presented as mean ± SD, *n* = 3. ^#^ *p* < 0.001 vs. the control group.

The cell membrane–mediated homologous targeting capability of JAPC/A@CM was first examined using confocal laser scanning microscopy (CLSM). As shown in Figure 3a, with the incubation of JAPC/A and JAPC/A@CM with A375 cells for 1, 3, and 6 h, the intracellular fluorescence intensity increased progressively over time, indicating efficient cellular internalization. Compared with the uncoated JAPC/A nanoparticles, the JAPC/A@CM group exhibited markedly higher uptake efficiency (Figure S7), confirming the enhanced homologous targeting effect conferred by the cancer cell membrane coating.

To assess the lysosomal escape capability of JAPC/A@CM, we examined its intracellular distribution at different incubation times using fluorescence imaging and quantitative analysis (Figure 3b). After 1 h of incubation, the red fluorescence of JAPC/A@CM showed strong colocalization with the green lysosomal signal, with a Pearson correlation coefficient of 0.83, indicating that the nanoparticles were primarily internalized and remained confined within lysosomes at this early stage. At 3 h, the degree of colocalization decreased, and by 6 h, the overlap between the two signals was further reduced, with the Pearson coefficient dropping to 0.43. These results indicate that JAPC/A@CM can escape from lysosomes into the cytosol, which is likely attributable to ROS‐induced oxidative damage and permeabilization of the lysosomal membrane [30]. Consistent with these observations, Bio‐TEM imaging provided direct visual evidence of cellular uptake and lysosomal escape (Figure 3c). At 0.5 h, JAPC/A@CM was observed in the process of cellular internalization, while at 6 h, the nanoparticles were clearly distributed within the cytoplasm, confirming successful lysosomal escape.

The CCK‐8 assay was used to evaluate the in vitro cytotoxicity of various nanoparticles. As shown in Figure 3d, JAPC or JAPC@CM alone exhibited only limited cytotoxicity for A375 cells. In contrast, L‐arginine supplementation or NIR irradiation markedly enhanced their therapeutic activity, likely due to the increased production of NO and RNS. Notably, the combined treatment with both L‐arginine and 808 nm NIR irradiation (JAPC/A@CM + L) resulted in the highest cytotoxicity, demonstrating a strong synergistic effect of NO‐enhanced PTT. Moreover, even at concentrations up to 100 µg mL^−^^1^, HUVECs maintained over 80% viability after 24 h of incubation (Figure 3e). This excellent biocompatibility may contribute to the relatively low GSH levels and the non‐acidic microenvironment in HUVECs compared with tumor cells, which limit the catalytic generation of ROS and RNS.

### 2.5 Multistimuli‐Activated Janus Nanomotor

**FIGURE 4:**
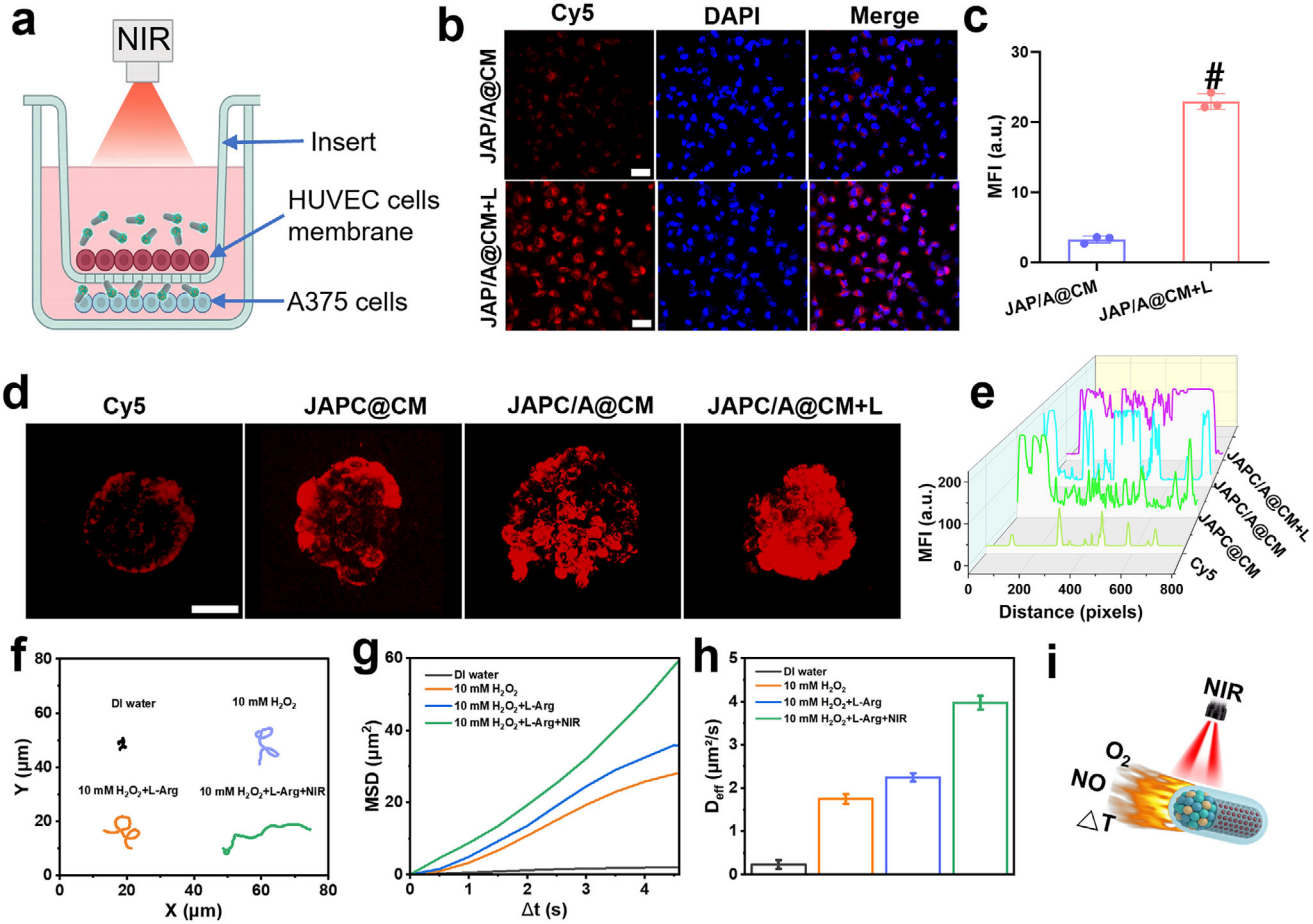
Motility behavior of JAPC/A@CM. (a) Schematic illustration of the transwell co‐culture system under NIR irradiation. (b) Fluorescence images and (c) quantitative analysis of cellular uptake of JAPC/A@CM in A375 cells after different treatments. Statistical analysis was performed using one‐way ANOVA followed by Tukey's multiple‐comparisons test. The data are presented as mean ± SD, *n* = 3. ^#^ *p* < 0.001 vs. the JAPC/A@CM group. (d) Fluorescence images of Cy5‐labeled nanoparticles in 3D tumor spheroids. Scale bar: 50 µm. (e) Line‐scan fluorescence intensity profiles across tumor spheroids. (f) Trajectories, (g) MSD curves, and (h) D_eff_ of nanomotors under different physicochemical stimulus conditions. (i) Schematic illustration of nanomotor propulsion driven by O_2_, NO, and NIR laser irradiation.

The asymmetric Janus structure not only endows JAPC/A@CM with synergistic functionalities but also enables autonomous directional motion. This multistimuli‐activated motile feature facilitates deeper penetration into tumor spheroids, thereby improving retention and promoting uniform NO distribution. To evaluate the penetration capability of JAPC/A@CM, we established a dual‐cell‐layer model mimicking the tumor vasculature–tumor tissue interface (Figure 4a). As shown in Figure 4b, in the absence of NIR irradiation, JAPC/A@CM exhibited limited penetration through the HUVEC–A375 cells co‐culture barrier. By contrast, upon NIR treatment, the JAPC/A@CM+L group displayed markedly stronger fluorescence signals in the lower layer of A375 cells (Figure 4c), indicating that NIR irradiation facilitates more efficient crossing of the vascular endothelial layer. A three‐dimensional tumor spheroid model further confirmed the enhanced intratumoral penetration (Figure 4d). Penetration depth showed a progressive rise from JAPC@CM to JAPC/A@CM and was highest in the JAPC/A@CM+L group. Notably, the JAPC/A@CM+L group exhibited robust signal intensity in the inner core of the spheroid, suggesting its ability to overcome the dense extracellular matrix and reach deeper tumor regions. Consistently, line‐scan analysis (Figure 4e) validated this observation, demonstrating that JAPC/A@CM+L maintained high fluorescence intensity across a larger depth range.

To elucidate the source of enhanced penetration, we further examined the motion behavior of the nanoparticles under various physicochemical stimulus conditions. As shown in Figure 4f and Figure S8, in the absence of catalytic substrates, the nanoparticles exhibited only in situ Brownian motion. The introduction of H_2_O_2_ markedly increased the particle trajectory, and the addition of L‐arginine further extended their motion. The combined presence of both substrates, together with NIR irradiation, resulted in the longest trajectory curves. This is because the AuPt_3_Cu nanozyme on the JAPC Janus side possesses CAT activity, which decomposes H_2_O_2_ to generate O_2_, thereby propelling autonomous locomotion. Upon the addition of L‐arginine, the AuPt_3_Cu nanozyme further catalyzes its reaction with H_2_O_2_ to generate NO, providing an additional chemical propulsion force. Under 808 nm NIR irradiation, the localized temperature rise of AuPt_3_Cu introduces a photothermal driving component, further enhancing nanoparticle motion. It was quantitatively verified by mean square displacement (MSD) analysis and the corresponding diffusion coefficient (D_eff_). Under the triple driving conditions of H_2_O_2_ + L‐arginine + NIR, JAPC nanoparticles exhibited the largest diffusion distance (Figure 4g) and highest diffusion rate (Figure 4h). Collectively, as shown in Figure 4i, these results demonstrate that JAPC/A@CM propels via a cooperative propulsion mechanism jointly driven by catalytic reactions and NIR‐mediated photothermal effects, thereby generating strong motile forces that facilitate deep tumor penetration and enhanced intratumoral delivery.

### 2.6 In Situ Self‐Cascade NO Generation

The intracellular delivery of L‐arginine alone results in limited NO production efficiency due to the hypoxic microenvironment and compensatory metabolic regulation of tumors. In contrast, the asymmetric architecture of JAPC/A@CM enables concurrent AuPt_3_Cu‐catalyzed oxidation of glucose to H_2_O_2_ and GSH‐responsive degradation of 2sMSN to release L‐arginine, thereby coupling nonenzymatic NO generation. Based on this design, the intracellular NO‐generating performance and mechanistic basis of JAPC/A@CM were first investigated.

**FIGURE 5:**
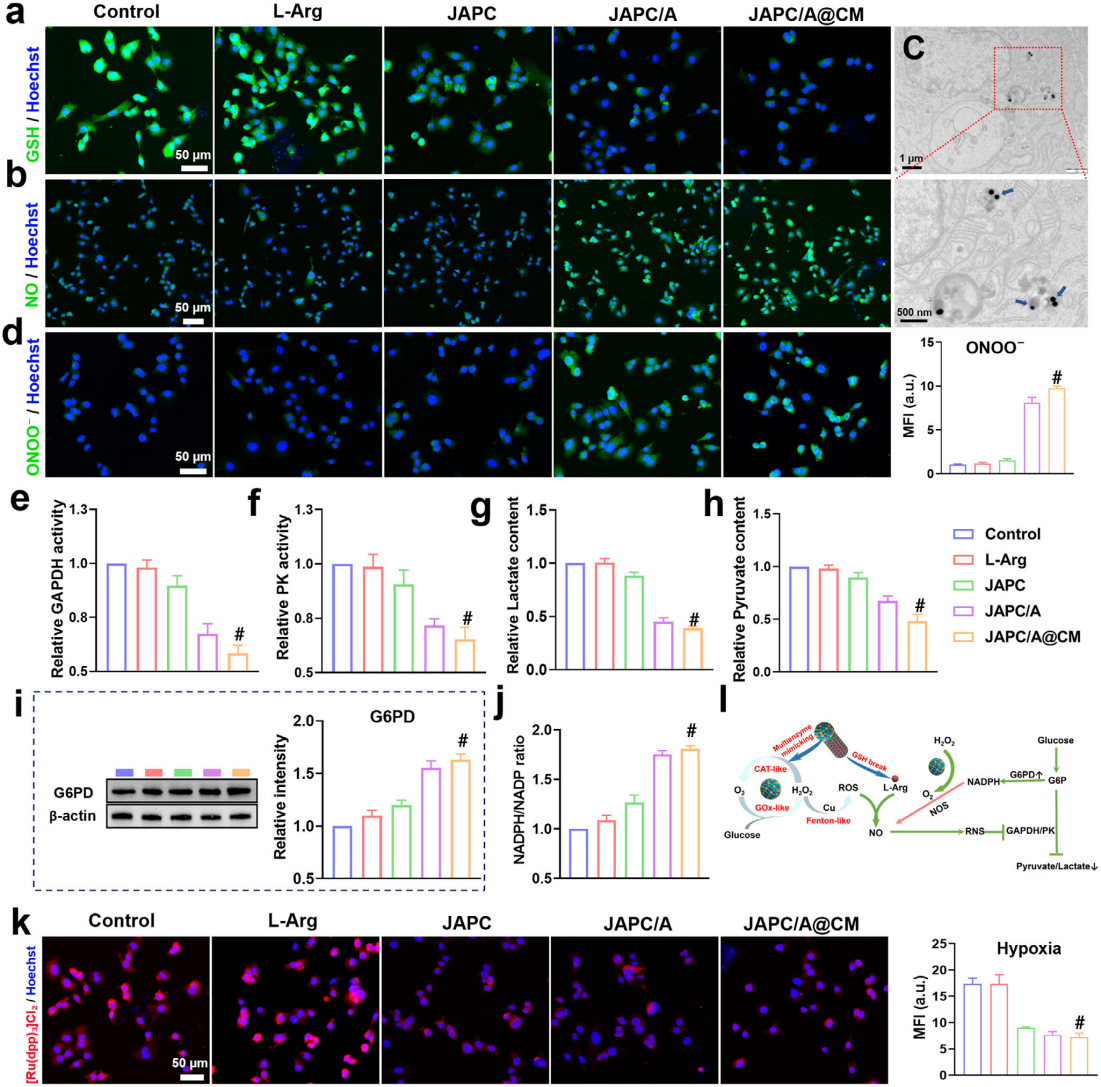
Self‐amplified cascade circuit for NO generation. (a) Fluorescence images of intracellular GSH levels stained with ThiolTracker Green and (b) intracellular NO generation detected using DAF‐FM DA. (c) Bio‐TEM images of cells incubated with JAPC/A@CM for 24 h. (d) Fluorescence images and quantitative analysis of intracellular ONOO^−^ generation detected using HKGreen‐4. (e) Quantitative analysis of relative GAPDH activity and (f) PK activity. (g) Quantitative analysis of relative intracellular lactate and (h) pyruvate content. (i) Western blot analysis of G6PD expression. (j) Quantitative analysis of relative intracellular NADPH/NADP^+^ ratio. (k) Fluorescence images and quantitative analysis of cellular hypoxia levels detected using [Ru(dpp)_3_]Cl_2_. (l) Schematic illustration of in situ cascade amplification of NO signaling mediated by JAPC/A@CM. Statistical analysis was performed using one‐way ANOVA followed by Tukey's multiple‐comparisons test. All data are presented as mean ± SD, *n* = 3. ^#^ *p* < 0.001 vs. the control group.

As shown in Figure 5a, intracellular GSH levels significantly decreased after JAPC treatment, and further dropped by more than 70% when L‐arginine was loaded (Figure S9). This effect results from the 2S‐bridged MSN framework, which consumes GSH during degradation and simultaneously releases L‐arginine. The released L‐arginine then serves as the substrate for NO cascade production to enhance oxidative stress, thereby further depleting GSH. Consistently, intracellular NO staining with the NO‐sensitive fluorescent probe DAF‐FM confirmed this process (Figure 5b). Control cells or cells treated with L‐arginine alone showed negligible NO signals, whereas the JAPC/A group exhibited a markedly enhanced NO fluorescence, with the MFI reaching sevenfold that of the control group (Figure S10), demonstrating that the NO cascade reaction indeed occurs intracellularly. In addition, compared to the JAPC/A group, the cell membrane coating group (JAPC/A@CM) improved nanoparticle uptake, resulting in an enhanced NO fluorescence signal. One limitation of the DAF‐FM assay is that, although it is widely used for intracellular NO detection, the fluorescence signal may also be affected by NO‐derived reactive nitrogen species or NO metabolites [31]. Thus, the DAF‐FM results should be interpreted as relative comparisons of NO levels across groups rather than absolute measurements of free NO. Because all samples were processed and analyzed under identical conditions, this limitation does not affect the comparative conclusions of this study. As shown in Figure 5c, Bio‐TEM imaging of JAPC/A@CM after 24 h incubation revealed substantial degradation of the MSN component (blue arrows), further supporting the GSH‐responsive generation of NO.

It has been widely reported that NO can further react with cellular ROS to form RNS, including peroxynitrite (ONOO^−^). ONOO^−^ is highly reactive and can modify thiol residues or metal cofactors in proteins through S‐nitrosylation, which often changes protein conformation and reduces enzyme activity [32, 33]. Several key glycolytic enzymes, such as glyceraldehyde‐3‐phosphate dehydrogenase (GAPDH) and pyruvate kinase (PK), contain reactive cysteine residues within their catalytic centers, which are extremely sensitive to RNS [34, 35]. Therefore, the glycolytic pathway is highly vulnerable to RNS‐mediated inhibition, owing to the nitration or S‐nitrosylation of its key catalytic enzymes. To further evaluate the impact of the Janus nanomotors on glycolytic metabolism, we first measured intracellular ONOO^−^ levels (Figure 5d). Cells treated with JAPC/A@CM showed a stronger ONOO^−^ signal than the control group, indicating active conversion of NO to RNS within cells. This is driven by the combined POD‐ and CAT‐like activities of AuPt_3_Cu, which supply both ROS and O_2_ required to promote the conversion of NO into RNS [27]. Furthermore, the enzymatic activities of GAPDH and PK (Figure 5e,f) were significantly reduced after treatment (*p* < 0.001), indicating the anticipated S‐nitrosylation of their catalytic cysteine residues by RNS. In line with these findings, the intracellular levels of lactate and pyruvate, which are recognized as metabolic hallmarks of high glycolytic flux, were also markedly decreased (Figure 5g,h), further demonstrating that glycolysis was strongly suppressed.

Upon inhibition of glycolysis and elevation of intracellular nitrosative stress, glucose flux was compensatorily redirected toward the pentose phosphate pathway (PPP) to sustain NADPH production and maintain redox homeostasis [36, 37, 38]. Indeed, cells treated with JAPC/A@CM displayed higher glucose‐6‐phosphate dehydrogenase (G6PD) expression (Figure 5i) and an elevated NADPH/NADP^+^ ratio (Figure 5j), indicating enhanced PPP flux.

In the TME, intracellular NO production via NOS‐dependent enzymatic pathways is often severely limited by hypoxia and insufficient cofactors [39, 40]. The AuPt_3_Cu component of JAPC/A@CM exhibits CAT‐like activity, enabling the conversion of endogenous H_2_O_2_ into O_2_ and thereby alleviating hypoxic stress. As shown in Figure 5k, the fluorescence intensity of the hypoxia probe in the JAPC/A@CM group was markedly lower than that in the control group, indicating effective relief of the hypoxic microenvironment. Consequently, together with the elevation of NADPH levels, the sustained oxygen availability provides favorable conditions for enhanced enzymatic NO production.

In summary, as illustrated in Figure 5l, upon cellular internalization, the asymmetric architecture of JAPC/A@CM simultaneously enables a triple enzyme–mimicking cascade that drives ROS generation and triggers GSH‐responsive L‐arginine release, leading to spatiotemporally controllable nonenzymatic NO production. Subsequently, derived RNS induces nitrative modifications of key glycolytic enzymes (GAPDH and PK), resulting in the suppression of glycolytic flux. The combined effects of glycolytic inhibition and oxidative stress promote compensatory upregulation of PPP, thereby enhancing intracellular NADPH production. Accumulated NADPH, together with dynamically generated O_2_, creates favorable conditions for reinforced NOS‐dependent enzymatic NO synthesis, establishing a NO‐centered positive metabolic feedback loop. Therefore, the JAPC/A@CM nanomotor functionally interconnects nonenzymatic and enzymatic NO‐generation pathways, enabling in situ cascade amplification of NO signaling.

### 2.7 Cascade NO‐Induced Mitochondrial Dysfunction

**FIGURE 6:**
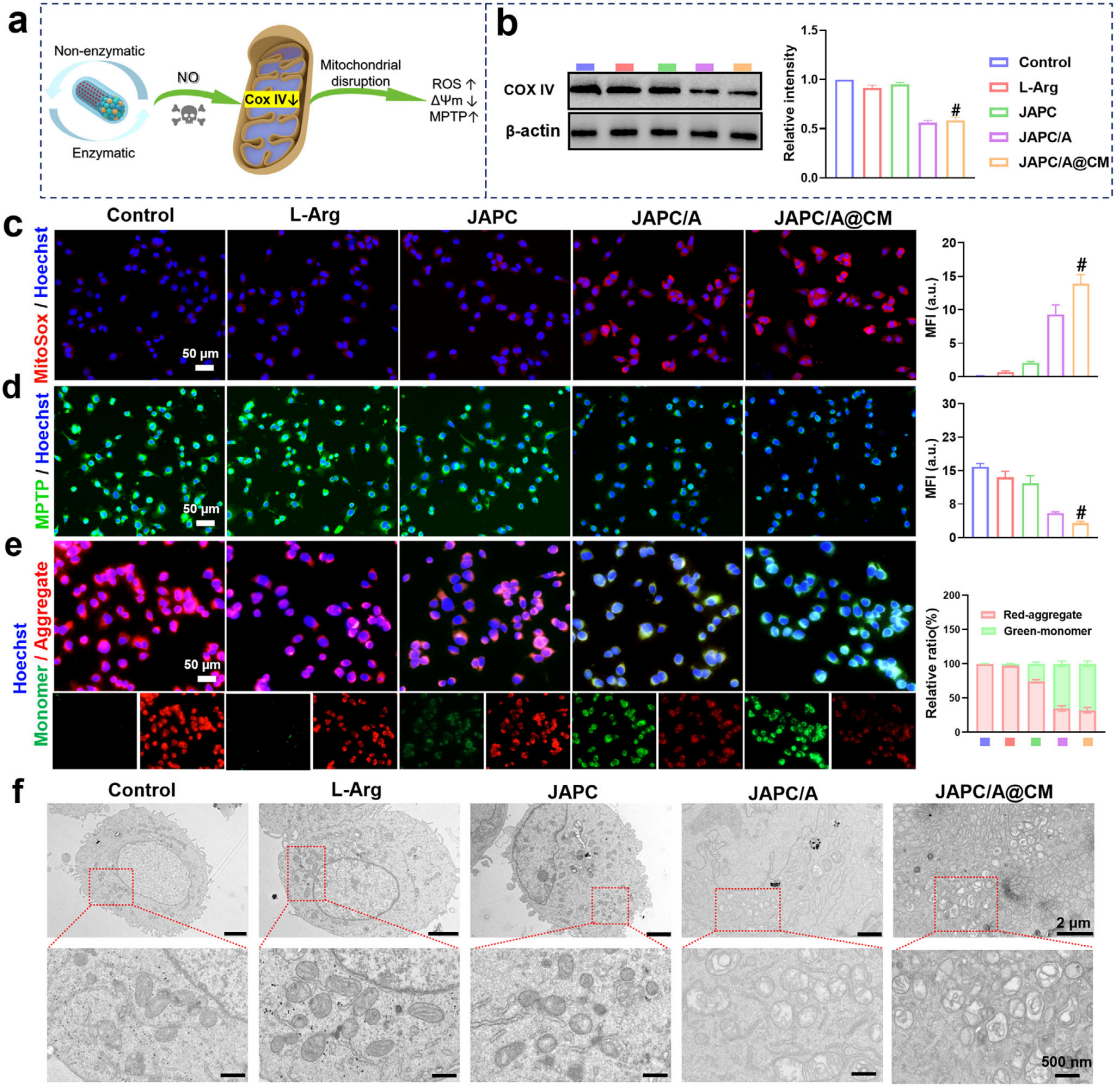
Mitochondrial dysfunction mediated by JAPC/A@CM. (a) Schematic illustration of NO‐induced mitochondrial dysfunction. (b) Western blot analysis of COX IV expression. (c) Fluorescence images of mitochondrial superoxide levels detected by MitoSOX Red staining. (d) MPTP opening detected using calcein‐AM/CoCl_2_ staining. (e) Mitochondrial membrane potential detected using the JC‐1 probe. (f) Bio‐TEM image of mitochondrial morphology. Statistical analysis was performed using one‐way ANOVA followed by Tukey's multiple‐comparisons test. All data are presented as mean ± SD, *n* = 3. ^#^ *p* < 0.001 vs. the control group.

High concentrations of NO are widely known to disrupt mitochondrial function [32]. As illustrated in Figure 6a, excess NO can competitively bind to mitochondrial cytochrome c oxidase (COX IV), thereby blocking electron transport, collapsing the mitochondrial membrane potential (ΔΨm), triggering ROS accumulation, and suppressing ATP production, ultimately leading to apoptosis. Based on this, the impact of JAPC/A@CM‐mediated amplified NO signaling on mitochondrial function was further systematically evaluated.

As shown in Figure 6b, Western blot results revealed a marked downregulation of COX IV in the JAPC/A@CM group, indicating impairment of mitochondrial respiratory chain integrity and function. This was further corroborated by MitoSOX staining (Figure 6c), which showed massive mitochondrial superoxide accumulation, as evidenced by a significant increase in red fluorescence MFI (*p* < 0.001), consistent with electron leakage resulting from disrupted electron transport. In addition, the mitochondrial permeability transition pore (MPTP) staining (Figure 6d) revealed a pronounced decrease in fluorescence intensity in the JAPC/A@CM group, indicating MPTP opening and early apoptosis. Accordingly, JC‐1 staining (Figure 6e) showed a marked reduction in red JC‐1 aggregates accompanied by an increase in green monomers, confirming severe mitochondrial ΔΨm collapse caused by MPTP opening. These mitochondrial dysfunction results were further validated by Bio‐TEM imaging. As shown in Figure 6f, mitochondria in the control and L‐arginine groups exhibited intact morphology with densely organized cristae. In contrast, mitochondria in the JAPC group showed partially blurred cristae, while the JAPC/A@CM group displayed severe mitochondrial swelling accompanied by pronounced cristae loss, indicating extensive mitochondrial structural damage. Together, the above results demonstrate that JAPC/A@CM induces mitochondrial disruption and structural damage through NO‐mediated respiratory chain inhibition.

### 2.8 Synergistic Effect of NO and mPTT

Based on the disruption of glycolysis and mitochondrial function, JAPC/A@CM simultaneously suppressed the two major energy metabolic pathways in tumor cells, resulting in a profound reduction in intracellular ATP production. As HSP90‐driven thermotolerance during PTT is highly ATP‐dependent [41], such metabolic stress is expected to impair cellular heat stress resistance. In addition, NO–induced S‐nitrosylation has been reported to promote conformational destabilization and degradation of HSP90 [42]. Therefore, the synergistic effect between the JAPC/A@CM‐mediated amplified NO and PTT was further investigated.

**FIGURE 7:**
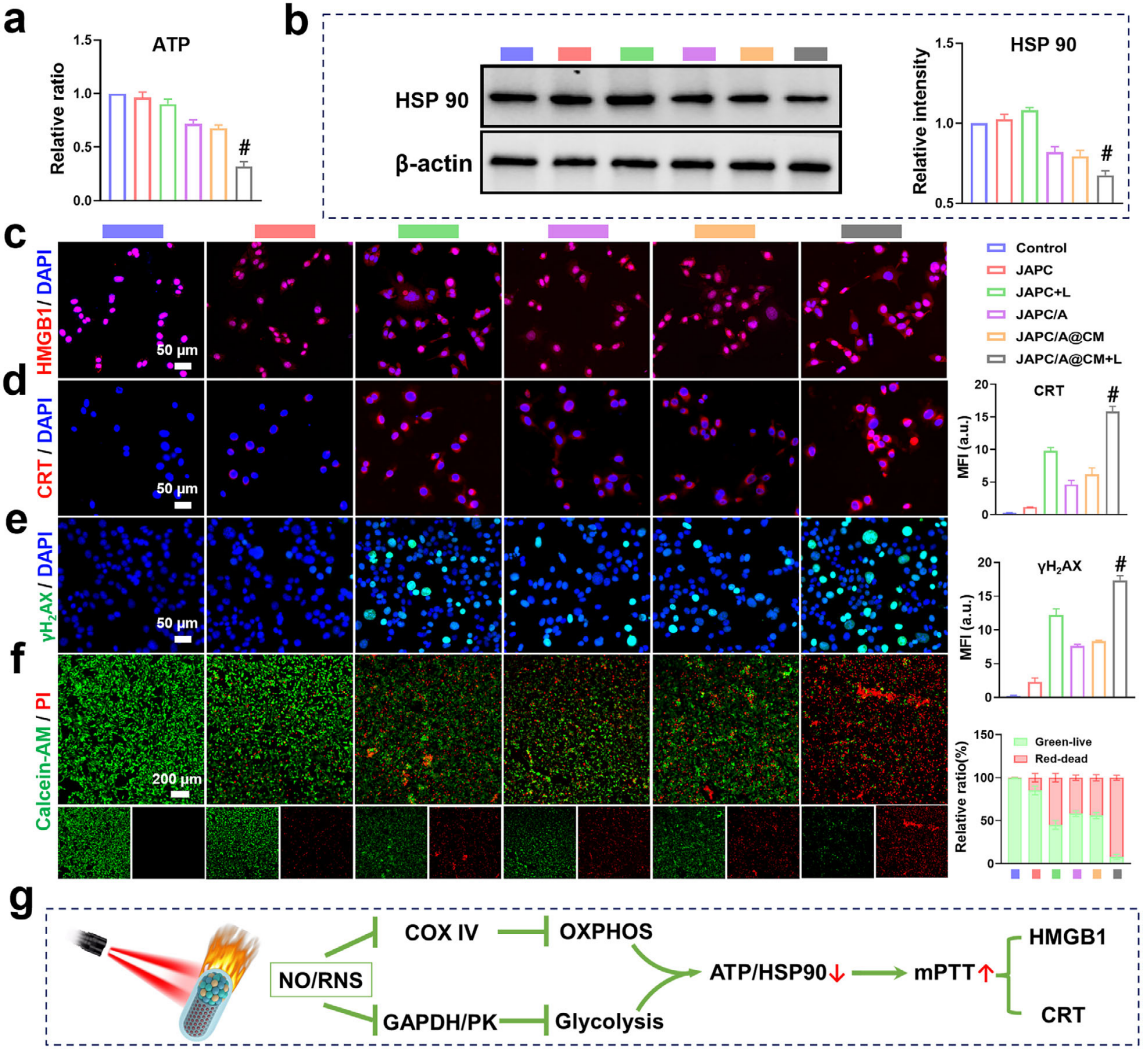
The synergistic effect of NO and mPTT. (a) Quantitative analysis of intracellular ATP levels in different treatment groups. (b) Western blot analysis of HSP90 expression. (c) Representative immunofluorescence images of HMGB1, (d) CRT, (e) γ‐H2AX after different treatments. (f) Live/Dead staining assay using calcein‐AM/PI to evaluate cell viability after different treatments. (g) Schematic illustration of JAPC/A@CM‐mediated OXPHOS and glycolysis suppression leading to the downregulation of ATP and HSP90, thereby enhancing the mPTT effect and inducing ICD. All scale bars: 50 µm. Statistical analysis was performed using one‐way ANOVA followed by Tukey's multiple‐comparisons test. All data are presented as mean ± SD, *n* = 3. ^#^ *p* < 0.001 vs. the control group.

As demonstrated in Figure 7a, JAPC/A@CM treatment led to a significant decrease in intracellular ATP levels, indicating that self‐reinforcing NO/RNS signaling amplifies energy depletion. Consistently, Figure 7b shows a marked downregulation of HSP90 expression, which may result from ATP insufficiency and protein destabilization induced by S‐nitrosylation. The reduction of HSP90 substantially increases tumor‐cell sensitivity to mPTT, thereby facilitating the exposure and release of immunogenic cell death (ICD) biomarkers. As shown in Figure 7c, the Janus nanomotor combined with laser irradiation (JAPC/A@CM + L group) induced robust release of high mobility group box 1 (HMGB1) from the nucleus into the extracellular space, indicating enhanced nuclear damage and activation of damage‐associated molecular patterns. Meanwhile, the JAPC/A@CM + L group markedly increased the cell‐surface exposure of calreticulin (CRT) (Figure 7d), an “eat‐me” signal that facilitates dendritic cell recognition and antigen presentation. γ‐H2AX staining further confirmed that JAPC/A@CM + L induced pronounced DNA double‐strand breaks (Figure 7e), suggesting a strong nuclear damage response. These results were further supported by Calcein‐AM/PI staining (Figure 7f). A substantial increase in PI‐positive cells accompanied by a marked decrease in viable cells was observed in the JAPC/A@CM + L group compared to either the L‐arginine–loaded group or the mPTT‐only group, demonstrating that the combination of NO therapy and mPTT markedly enhances tumor‐cell killing efficiency. These results suggest that NO‐mediated HSP90 downregulation may weaken tumor‐cell thermotolerance and increase sensitivity to mPTT‐induced proteotoxic stress [43, 44], thereby contributing to enhanced therapeutic efficacy.

Collectively, as shown in Figure 7g, JAPC/A@CM suppresses both mitochondrial respiration and glycolytic metabolism, inducing energy exhaustion and weakening HSP90‐mediated chemoprotective mechanisms, thereby sensitizing tumor cells to mPTT. The self‐amplifying NO/RNS combined with PTT, effectively triggers HMGB1 release, CRT exposure, DNA damage, and subsequent cell death, achieving potent activation of ICD.

### 2.9 In Vivo Distribution and Photothermal Evaluation

**FIGURE 8:**
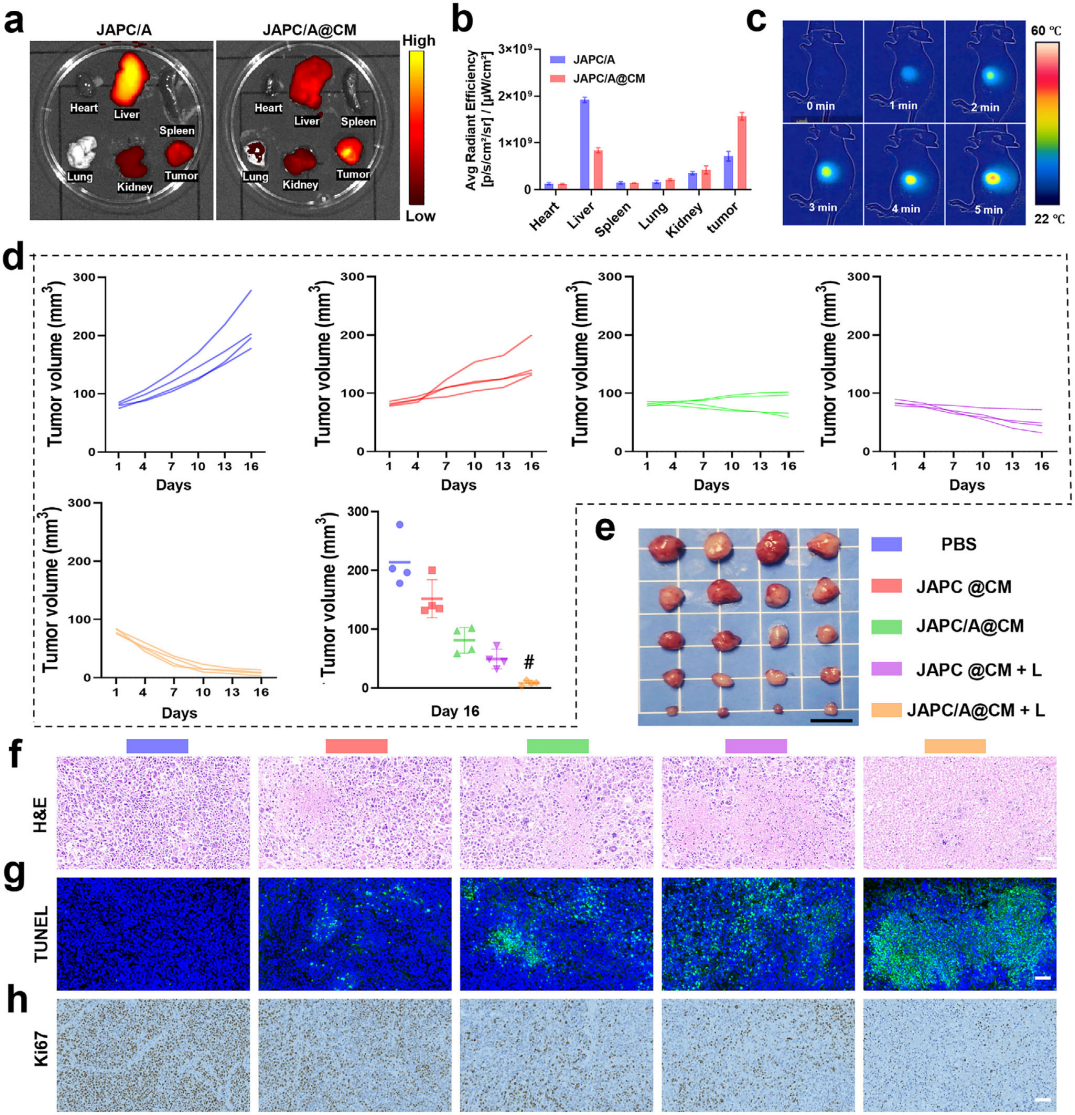
In vivo antitumor efficacy and histological analyses. (a) Fluorescence images and (b) quantitative analysis of major organs and tumors harvested from tumor‐bearing mice 24 h after intravenous injection of JAPC/A or JAPC/A@CM. (c) Infrared thermal images of tumor‐bearing mice recorded at different time points during 808 nm laser irradiation. (d) Tumor growth curves and corresponding tumor volumes of mice in different groups. (e) Photographs of excised tumors after 16 days of treatment. The scale bars: 1 cm. (f) Representative images of H&E, (g) TUNEL, and (h) Ki67 staining from different groups. All scale bars: 50 µm. Statistical analysis was performed using one‐way ANOVA followed by Tukey's multiple‐comparisons test. All data are presented as mean ± SD, *n* = 4. ^#^ *p* < 0.001 vs. the PBS group.

To examine the in vivo distribution of the nanomaterials, major organs and tumors from tumor‐bearing mice were subjected to fluorescence imaging after intravenous injection (Figure 8a). Compared to JAPC/A, the cell membrane‐coated JAPC/A@CM nanoparticles showed stronger fluorescence accumulation in tumors and relatively lower signals in the liver. Quantitative analysis (Figure 8b) confirmed that the tumor accumulation of JAPC/A@CM was approximately twofold higher than that of JAPC, indicating improved biodistribution and tumor targeting. This enhanced tumor enrichment is attributed to the cell membrane–driven homologous targeting, which mediates the in vivo tumor‐homing behavior [45]. Subsequently, the photothermal performance of JAPC/A@CM in vivo was assessed by infrared thermal imaging. As shown in Figure 8c and Figure S11, under 808 nm irradiation, the tumor region in mice treated with JAPC/A@CM rapidly heated to nearly 60°C within 5 min, indicating its efficient photothermal conversion sufficient to support mPTT.

### 2.10 In Vivo Antitumor Efficacy

The antitumor efficacy of the Janus nanomotor in vivo was evaluated by a subcutaneous tumor model. Tumor‐bearing mice were randomly divided into five treatment groups: PBS, JAPC@CM, JAPC/A@CM, JAPC@CM + L, and JAPC/A@CM + L. Treatments were administered every three days, and tumors were harvested on day 16. As shown in Figure 8d, the tumor growth curves gradually diverged among the treatment groups. Tumors in the PBS group expanded rapidly, whereas JAPC@CM slowed tumor progression, which may be related to its triple enzyme‐like activity that induces oxidative stress. JAPC/A@CM and JAPC@CM + L groups both showed moderate tumor suppression, reflecting the limited therapeutic efficacy of NO/RNS generation and photothermal therapy, respectively. Notably, the combination treatment (JAPC/A@CM + L group) yielded the smallest tumor volume at the endpoint, with a tumor inhibition rate of 95%, demonstrating a pronounced synergistic antitumor effect. Excised tumors (Figure 8e) were consistent with the tumor growth curves, further demonstrating the superior antitumor efficacy of the combination treatment.

In addition, there were no significant changes in body weight (Figure S12) observed across all groups throughout the treatment period, and H&E staining of major organs (liver, kidney, heart, lung, and spleen) showed no apparent pathological abnormalities (Figure S13). These results indicate that JAPC/A@CM possesses good biocompatibility at the administered therapeutic dose.

Histological analysis was further conducted to verify the antitumor mechanism of this Janus nanoplatform. H&E staining (Figure 8f) revealed that either NO‐based gas therapy or mPTT alone caused visible tumor tissue damage, whereas the combined treatment group exhibited a substantially expanded apoptosis area, indicating a strong synergistic interaction between the two therapeutic modalities. Moreover, TUNEL staining (Figure 8g) and Ki67 immunofluorescence (Figure 8h) showed that the combination therapy group induced the most prominent apoptotic signal, accompanied by a pronounced decrease in tumor cell proliferation, further confirming that the integrated strategy effectively suppressed tumor growth in vivo. Collectively, these results demonstrate that the combination of NO therapy and mPTT induced significant tumor cell death and inhibited tumor proliferation in vivo.

In addition, RNS probe staining (Figure S14) showed the strongest fluorescence signals in tumor tissues from the JAPC/A@CM and JAPC/A@CM+L groups, indicating that the nanoplatform can trigger cooperative enzymatic reactions with L‐arginine in vivo and markedly accelerate NO/RNS accumulation, which is consistent with the in vitro observations. HSP90 immunostaining (Figure S15) further revealed a marked reduction in protein expression within the combination group, suggesting that RNS‐induced oxidative stress and energy depletion suppressed the heat‐shock protective mechanism of tumor cells, thereby amplifying the therapeutic efficacy of mPTT.

To determine whether ICD occurred in vivo, CRT and HMGB1 immunofluorescence staining were performed. As shown in Figure S16, JAPC/A@CM+L treatment led to a strong elevation of CRT signals, indicating that the “eat‐me” signal was exposed on the tumor cell surface, facilitating dendritic cell recognition and antigen uptake. Meanwhile, HMGB1 staining displayed evident extracellular release, demonstrating typical DAMPs release from tumor cells and further confirming the activation of ICD. The above results demonstrate that JAPC/A@CM induces sustained RNS generation and suppresses HSP90 expression in vivo, thereby enhancing mPTT and promoting CRT exposure and HMGB1 release, ultimately activating the ICD pathway. These results indicate that the synergistic action between NO/RNS and mPTT not only directly induces tumor cell death but also substantially increases tumor immunogenicity, providing powerful therapeutic potential for this nanoplatform.

Although nude mice are deficient in mature T cells due to thymic developmental defects and therefore lack a fully functional T cell‐mediated adaptive immune response, they still retain innate and myeloid immune components, including macrophages, dendritic cell‐like antigen‐presenting cells, and other myeloid immune cells [46]. Therefore, to further evaluate local immune activation in the tumor microenvironment, we examined the expression of CD80 and CD86, two costimulatory molecules associated with activated antigen‐presenting/myeloid immune cells, in tumor tissues. Immunofluorescence staining (Figure S17) showed that CD80 and CD86 signals were markedly increased in the treated tumors compared with the PBS group, suggesting enhanced local activation of antigen‐presenting/myeloid immune cells in the tumor microenvironment. Nevertheless, these findings should be interpreted within the context of the A375 xenograft model established in BALB/c nude mice. Although this model is useful for evaluating antitumor efficacy and local innate/myeloid immune activation, its T cell‐deficient nature limits assessment of the complete downstream immune cascade triggered by immunogenic cell death, including dendritic cell maturation, T‐cell priming and activation, systemic antitumor immunity, and immune memory. Therefore, future studies using immunocompetent syngeneic tumor models are warranted to further validate ICD‐mediated immune activation by evaluating dendritic cell maturation markers, together with T‐cell responses, tumor rechallenge, and metastasis models.

Collectively, NO–based cancer therapy holds immense promise but is fundamentally constrained by two intrinsic challenges: the insufficient intratumoral penetration of exogenous donors and the inability to sustain a therapeutically relevant NO flux against the tumor's metabolic adaptability. Current strategies, which rely predominantly on passive accumulation and static substrate supplementation, fail to overcome the spatial heterogeneity of the tumor microenvironment, rendering NO signaling transient, spatially discontinuous, and therapeutically suboptimal. In this work, this Janus nanomotor can actively penetrate deep tumor regions under multiple cues (H_2_O_2_, L‐arginine, and NIR irradiation), thereby overcoming perivascular accumulation and achieving uniform intratumoral distribution. This asymmetric architecture allows AuPt_3_Cu‐mediated catalysis to sustain an H_2_O_2_/O_2_/ROS production cycle, while the glutathione‐responsive 2sMSN shell continuously releases L‐arginine as a substrate, thereby coupling H_2_O_2_‐mediated L‐arginine oxidation. These synergistic functions endowed by the Janus structure collectively enable precise spatiotemporal modulation of NO generation. Furthermore, the uniformly distributed NO induces nitrosative stress and glycolysis inhibition, thereby triggering compensatory upregulation of PPP and promoting NADPH generation. The accumulated NADPH, together with dynamically generated O_2_, reinforces NOS‐dependent NO formation, establishing a NO‐centered positive metabolic loop that maintains intratumoral NO flux above the therapeutic threshold. More importantly, the self‐cascade amplification of NO downregulates HSP90 expression, thereby enhancing mPTT and promoting immunogenic cell death, ultimately achieving potent melanoma eradication.

Notably, NO‐mediated modulation of tumor transport barriers may further cooperate with the asymmetric structure‐driven active movement of JAPC/A@CM. On the one hand, the asymmetric Janus structure endows the nanoplatform with active nanomotor‐like movement, allowing it to move beyond the tumor periphery and achieve deeper tumor infiltration. On the other hand, NO generated in the tumor microenvironment may further facilitate nanoparticle transport by regulating biological barriers associated with tumor delivery. Previous studies have shown that NO can normalize tumor vessels and improve the intratumoral delivery of albumin‐bound paclitaxel nanoparticles [47], induce stromal depletion to enhance nanoparticle penetration in pancreatic tumors [48], and promote nanoparticle transport across tumor vascular basement membranes by opening endothelial junctions and remodeling the local basement membrane barrier [49]. These studies suggest that NO can act not only as a therapeutic gas molecule but also as a regulator of tumor vascular and stromal transport barriers. Therefore, in the present study, JAPC/A@CM integrates asymmetric structure‐mediated active movement with NO generation, enabling enhanced tumor penetration while simultaneously producing NO‐mediated therapeutic effects. This structure–microenvironment synergistic mechanism further supports the therapeutic potential of JAPC/A@CM for melanoma treatment.

Overall, this multistimuli‐activated Janus nanomotor leverages an “asymmetric structure‐driven functional synergy” strategy to orchestrate autonomous motility, deep tumor penetration, and spatiotemporally coordinated catalytic functions, thereby enabling controllable, sustained, and uniform NO generation within the TME.

## 3 Conclusion

In conclusion, this multistimuli‐activated Janus nanomotor enables controllable, sustained, and spatially uniform NO generation within the tumor microenvironment. By coupling AuPt_3_Cu‐mediated ROS cycling with GSH‐responsive L‐arginine release, the Janus nanomotor integrates nonenzymatic and enzymatic pathways to establish a self‐reinforcing NO production loop. The amplified NO signal further cooperates with mPTT to induce immunogenic cell death, ultimately resulting in potent melanoma eradication. Collectively, this work establishes an “asymmetric structure‐driven functional synergy” strategy for programmable intratumoral gaseous signaling, offering a novel conceptual framework for gas‐based combination therapy.

## 4 Experimental Section

### 4.1 Materials

All the chemicals were of analytical grade and used without further purification. Chloroauric acid trihydrate (HAuCl_4_·3H_2_O), Chloroplatinic acid hexahydrate (H_2_PtCl_6_·6H_2_O), ascorbic acid, CTAB, Copper chloride dihydrate, Bis(triethoxysilylpropyl) disulfide (BTES), methyl alcohol, tetraethyl orthosilicate (TEOS), 5,5'‐Dithiobis‐(2‐Nitrobenzoic Acid) (DTNB), Cy5, L‐arginine, and GSH were purchased from Sigma–Aldrich (USA). HSP90, COX IV, iNOS, CRT, and HMGB1 antibodies were obtained from Proteintech (Wuhan, China). NIR 808 nm laser was obtained from Xi'an Lazer Electronic Technology Co., Ltd. (China).

### 4.2 Characterization

Particle size and zeta potential were measured using Zetasizer Nano (Malvern Panalytical, UK). The morphology of the nanoparticles was observed by transmission electron microscopy (JEM‐1400 Plus, JEOL, Japan). Cell imaging experiments were carried out using a Zeiss LSM 880 confocal microscope with Airyscan and an inverted fluorescence microscope (Axio Observer, Zeiss, Germany). UV–vis absorption spectra were collected using a SpectraMax Plus spectrophotometer (Molecular Devices, USA). X‐ray diffraction patterns were obtained on a Bruker D8 Advance diffractometer (Bruker, Germany). Surface chemical properties were analyzed by x‐ray photoelectron spectroscopy (ESCALAB Xi+, Thermo Fisher Scientific, USA) and Fourier transform infrared spectroscopy (PerkinElmer, USA).

### 4.3 Synthesis of AuPt_3_Cu Nanozymes

AuPt_3_Cu nanozymes were synthesized via a one‐pot reduction method. Briefly, aqueous solutions of 0.296 mg of HAuCl_4_·3H_2_O, 1.17 mg of H_2_PtCl_6_·6H_2_O, and 0.170 mg of CuCl_2_·2H_2_O were mixed under vigorous stirring, followed by the addition of L‐ascorbic acid (70 mmol·L^‒1^) as a reducing agent. The reaction was maintained at room temperature for 4 h to ensure complete reduction. The resulting AuPt_3_Cu nanoparticles were collected by centrifugation, washed several times with DI water, and redispersed for further use. AuPt_3_ nanozymes were synthesized following the same procedure as described above, except that CuCl_2_·2H_2_O was not added.

### 4.4 Fabrication of Janus AuPt_3_Cu@2sMSN

Janus AuPt_3_Cu@2sMSN nanoparticles were prepared via an asymmetric sol–gel coating process. Briefly, AuPt_3_Cu nanoparticles pre‐dispersed in ethanol were introduced into an aqueous solution containing CTAB (5.5 mm), followed by the addition of ammonia solution (0.5 m) and continuous stirring for 30 min. Subsequently, TEOS and BTES (2 mm) were added to form the mesoporous silica framework. The resulting JAPC nanoparticles were collected and thoroughly washed to remove residual reactants.

### 4.5 Fabrication of JAPC/A

10 mg JAPC nanoparticles were dispersed in an aqueous L‐arginine solution (5 mg mL^−1^) and incubated under gentle stirring overnight. After loading, the nanoparticles were collected by centrifugation and washed to remove excess L‐arginine.

### 4.6 Fabrication of JAPC/A@CM

A375 cells were harvested and subjected to hypotonic lysis and differential centrifugation to isolate cell membranes. The purified membranes were then mixed with JAPC/A nanoparticles and co‐extruded through polycarbonate membranes to yield cell membrane–coated nanoparticles. The resulting particles were collected and stored at 4°C for cell experiments. JAPC/A‐Cy5@CM was synthesized following the same procedure as described above, except that Cy5 was added during the process of AuPt_3_Cu@2sMSN.

### 4.7 GSH‐Responsive Degradation Assay

GSH‐responsive degradation behavior of JAPC/A@CM was evaluated by incubating the nanoparticles in DI water or DI water containing 10 mm GSH at pH 6.5 for 72 h. Structural changes were monitored by TEM to assess the stability of the Janus architecture and the degradation of the disulfide‐bridged mesoporous silica shell.

### 4.8 Multienzyme‐Mimicking Activity

APC indicates AuPt_3_Cu nanozyme, JAPC indicates Janus AuPt_3_Cu@2sMSN, JAPC/A indicates L‐arginine‐loaded JAPC, JAPC/A@CM indicates cell membrane–coated JAPC/A, and L indicates irradiation with an 808 nm NIR laser. Glucose consumption was quantified using an o‐toluidine–based glucose assay kit (Beyotime, Shanghai, China). In this assay, o‐toluidine reacts with glucose to form a blue–green Schiff base, which exhibits a characteristic absorbance maximum at 630 nm. Briefly, JAPC/A at concentrations of 50, 100, and 200 µg mL^−1^ was incubated with glucose solution (1 mg mL^−1^) at 37°C for 24 h. After incubation, the reaction mixtures were combined with the o‐toluidine reagent and heated in a metal bath at 95°C for 10 min, followed by rapid cooling in an ice bath to 4°C. The resulting solutions were transferred to cuvettes, and the absorbance was recorded at 630 nm using a UV–vis spectrophotometer. In addition, for the 200 µg mL^−1^ JAPC/A group, changes in solution pH resulting from gluconic acid formation were continuously monitored using a calibrated pH meter.

The generation of H_2_O_2_ was determined using a commercial hydrogen peroxide assay kit (Beyotime, China). JAPC/A at concentrations of 50, 100, and 200 µg mL^−1^ were incubated with glucose solution (1 mg mL^−1^) at room temperature for 24 h. Subsequently, ferrous ions were added to the reaction system, followed by incubation at 30°C for 30 min. The resulting solutions were transferred to cuvettes, and the absorbance was recorded at 560 nm using a UV–vis spectrophotometer.

The CAT‐mimicking activity of the samples was assessed by examining oxygen generation. In a typical procedure, JAPC/A suspension (2 mg mL^−1^) was mixed with a hydrogen peroxide solution in 5 mL of deionized water and incubated for 20 min. The dissolved oxygen level in the reaction system was subsequently quantified using a dissolved oxygen analyzer (JPBJ‐609L, Leici, China).

The POD‐like activity was investigated using the TMB–based colorimetric assay. In brief, 20, 40, and 60 µL of JAPC/A suspension (2 mg mL^−1^) were individually added to an acetate buffer solution (HAc–NaAc, pH 6.5) containing TMB (16 mm) and hydrogen peroxide. After incubation, aliquots of the reaction mixtures were collected and subjected to UV–vis spectroscopic analysis.

The GSH consumption capability of JAPC/A nanoparticles was evaluated using the DTNB method. Briefly, JAPC/A nanoparticles at concentrations of 100 and 200 µg mL^−1^ were co‐incubated with GSH solution (10 mm) in buffer at pH 6.5 for 24 h. After incubation, the reaction mixtures were treated with DTNB reagent, which reacts with the remaining reduced GSH to generate 2‐nitro‐5‐thiobenzoic acid, producing a yellow‐colored product. The absorbance of the resulting solutions was measured by UV–vis spectroscopy at 412 nm.

### 4.9 NO and RNS Detection

NO generation was evaluated using a fluorescence probe–based method with DAF‐FM. Briefly, the JAPC/A nanoparticles were dispersed in buffer solution and mixed with DAF‐FM, H_2_O_2_ (10 mm), and L‐arginine (10 mm). The mixtures were incubated for different time intervals (10, 20, and 30 min). The fluorescence intensity was then measured using a fluorescence spectrophotometer. RNS generation was further assessed using an ONOO^−^ probe, Rhodamine B hydrazide (BBoxiProbe O72, Beibo, Shanghai, China).

### 4.10 Photothermal Performance

To evaluate the photothermal characteristics of JAPC/A@CM, aqueous dispersions of the nanoparticles were prepared at concentrations of 0.1, 0.25, and 0.5 mg mL^−^^1^ in 2 mL polypropylene tubes. Samples at each concentration were subjected to 808 nm NIR laser irradiation at power densities of 0.5, 0.75, and 1.0 W cm^−^^2^ for 5 min. Deionized water was treated under identical conditions and served as the negative control. Temperature changes during laser exposure were continuously monitored using an infrared thermal imaging system (FLIR E76, USA). Furthermore, the photothermal stability of JAPC/A@CM was investigated through three consecutive laser ON/OFF irradiation cycles under identical irradiation parameters.

### 4.11 Cell Culture

A375 and HUVEC cell lines were obtained from ATCC and cultured according to the supplier's protocols. A375 cells were cultured in Dulbecco's Modified Eagle Medium (DMEM), while HUVECs were cultured in EGM‐2 medium. The media were supplemented with 10% fetal bovine serum (FBS), 1% penicillin–streptomycin (PS), 1% HEPES, and 1% non‐essential amino acids (NEAA). Cells were cultured in a humidified incubator at 37°C with 5% CO_2_, and the culture media were replaced every two days. Mycoplasma detection was performed using the LookOut Mycoplasma PCR Detection Kit (Sigma–Aldrich, MP0035) according to the manufacturer's instructions. All cells used in the experiments were confirmed to be mycoplasma‐free. For cellular experiments, cells were assigned to the following treatment groups to evaluate cellular behavior: PBS, L‐arginine, JAPC, JAPC/A, JAPC/A@CM, 808 nm laser irradiation (L), JAPC/A@CM + L, and JAPC + L. APC indicates AuPt_3_Cu nanozyme, JAPC indicates Janus AuPt_3_Cu@2sMSN, JAPC/A indicates L‐arginine‐loaded JAPC, JAPC/A@CM indicates cell membrane–coated JAPC/A, and L indicates irradiation with an 808 nm NIR laser.

### 4.12 Cellular Uptake

A total of 20,000 cells were seeded in confocal dishes and incubated for 24 h to ensure adhesion. The culture medium was then replaced with fresh medium containing either JAPC/A‐Cy5 or JAPC/A‐Cy5@CM nanoparticles, followed by incubation for 1, 3, or 6 h. After incubation, the cells were washed with PBS, fixed, and imaged by CLSM to evaluate intracellular nanoparticle uptake.

### 4.13 Lysosomal Escape Assay

A total of 20 000 cells were seeded in confocal dishes and incubated for 24 h to ensure adhesion. The cells were then incubated with JAPC/A‐Cy5@CM nanoparticles for 1, 3, or 6 h. After incubation, the cells were washed with PBS and stained with LysoTracker Green (Invitrogen, Thermo Fisher Scientific) for 20 min. The cells were subsequently imaged by CLSM.

### 4.14 Cytotoxicity

A375 and HUVEC cells were seeded into 96‐well plates and incubated for 24 h to ensure adhesion. After 24 h, the culture medium was replaced with fresh medium containing different treatments, and the cells were further incubated for 24 h. Subsequently, the medium was replaced with fresh medium supplemented with 10% CCK‐8 reagent. After an additional 3 h of incubation, the absorbance at 450 nm was measured using a microplate reader.

### 4.15 Bio‐TEM

Following the corresponding treatment, the cells were washed with PBS and fixed with 2.5% glutaraldehyde at 4°C. The cells were subsequently post‐fixed with 1% osmium tetroxide, dehydrated, and embedded in epoxy resin to prepare the ultrathin sections. After uranyl acetate and lead citrate staining, a transmission electron microscope was used to observe intracellular nanoparticle localization and ultrastructural features.

### 4.16 Calcein‐AM Staining

A375 cells were seeded in confocal dishes at a density of 3 × 10^5^ cells per dish and subjected to different treatments. After washing with PBS, the cells were incubated with fresh medium containing Calcein‐AM (Beyotime, Shanghai, China) and propidium iodide (Beyotime, Shanghai, China) for 20 min. The cells were then imaged by CLSM.

### 4.17 Motion Study

The motility behavior of nanoparticles under different conditions was recorded using an inverted optical microscope. For the NIR irradiation group, an 808 nm laser was applied. Hydrogen peroxide and L‐arginine were added where indicated. Time‐lapse images were acquired at fixed intervals to monitor the real‐time movement of individual nanoparticles. The acquired image sequences were analyzed using ImageJ software with the Manual Tracking plugin. Individual nanoparticle trajectories were tracked frame by frame, and the resulting trajectory data were used to calculate the MSD and effective diffusion coefficient (D_eff_). For 2D trajectory analysis, MSD was calculated as MSD = (Δx)^2^ + (Δy)^2^, and D_eff_ was calculated as D_eff_ = MSD / (4Δt), where Δx and Δy represent the particle displacement in the x and y directions, respectively, and Δt represents the time interval.

### 4.18 Intracellular Glutathione Detection

A375 cells (3 × 10^5^) were seeded in confocal dishes and cultured for 24 h to ensure adhesion. The culture medium was then replaced, and the cells were treated with nanoparticles from different groups for an additional 24 h. After treatment, the medium was replaced with fresh medium containing ThiolTracker Violet (Invitrogen, Thermo Fisher Scientific) at a final concentration of 10 µM, followed by incubation for 24 h. The cells were subsequently imaged by CLSM to evaluate intracellular GSH depletion.

### 4.19 Intracellular Oxygen Detection

A375 cells (3 × 10^5^) were seeded in confocal dishes and cultured for 24 h to ensure adhesion. After washing with PBS, the cells were treated with different nanoparticle formulations and further incubated for 8 h. Subsequently, [Ru(dpp)_3_]Cl_2_ was added, and the cells were incubated. Fluorescence imaging was then performed to assess intracellular oxygen levels.

### 4.20 Intracellular NO Detection

A375 cells (3 × 10^5^) were seeded in confocal dishes and cultured for 24 h to ensure adhesion. The cells were then treated with different nano‐formulations in the presence of DAF‐FM DA (Beyotime, Shanghai, China). After treatment, the cells were rinsed twice with PBS and imaged by CLSM to evaluate intracellular NO generation.

### 4.21 Intracellular ONOO^−^ Detection

A375 cells (3 × 10^5^) were seeded in confocal dishes and cultured for 24 h to ensure adhesion. Following treatment, the medium was replaced with 1 mL of fresh medium containing an ONOO^−^ fluorescent probe (HKGreen‐4I, MedChemExpress, Cat. No. HY‐D1148). The cells were subsequently stained with Hoechst 33342 and imaged by CLSM.

### 4.22 GAPDH and PK Enzymatic Activity Assay

GAPDH enzymatic activity was measured using a GAPDH Activity Assay Kit (Invitrogen, Thermo Fisher Scientific). After different treatments, cells were washed with PBS and lysed with the kit‐provided lysis buffer on ice. Lysates were centrifuged at 12 000 × g for 10 min at 4°C to remove insoluble debris, and the supernatants were collected. Protein concentration was determined using a BCA Protein Assay Kit (Thermo Fisher Scientific) to normalize input. For each sample, equal amounts of protein were mixed with the GAPDH reaction mixture prepared according to the manufacturer's instructions. The reaction was incubated at 37°C for 20 min. GAPDH activity was measured based on the increase in fluorescence as specified by the kit, using a microplate reader. Enzymatic activity was normalized proportionally within each group.

Cells were collected and lysed in PK assay buffer provided by the assay kit (Abcam, Cambridge, UK), and lysates were clarified by centrifugation at 12 000 × g for 10 min at 4°C. Supernatants were used for the assay. A reaction mixture containing phosphoenolpyruvate, ADP, and coupled detection reagents was prepared as directed. Samples were incubated with the reaction mixture in a 96‐well plate for 15 min, and absorbance was monitored by a microplate reader at 570 nm. Enzymatic activity was normalized proportionally within each group.

### 4.23 Intracellular Lactate Measurement

Intracellular lactate levels were quantified using a Lactate Assay Kit (Beyotime, Shanghai, China). After treatments, cells were washed with PBS and harvested. Cells were lysed using the extraction buffer supplied with the kit, and lysates were centrifuged at 2500 × g for 10 min at 4°C to collect supernatants. Sample supernatants were mixed with the kit reagents to allow enzymatic conversion of lactate to a detectable product. After incubating at 37°C for 30 min, absorbance was measured using a microplate reader. Lactate levels were calculated based on the standard curve and normalized proportionally within each group.

### 4.24 Intracellular Pyruvate Measurement

Intracellular pyruvate was measured using a Pyruvate Assay Kit (Abcam, Cambridge, UK). Cells were washed with PBS and lysed in the assay buffer. Lysates were centrifuged at 12 000 × g for 5 min at 4°C, and supernatants were collected. Samples were incubated with the kit's reaction mix containing enzyme and detection components at room temperature for 30 min. A microplate reader was used for signal detection at 570 nm.

### 4.25 Intracellular NADPH Measurement

Intracellular NADPH levels were measured using an NADP/NADPH Quantitation Kit (WST‐8 method, Beyotime Biotechnology, Shanghai, China) according to the manufacturer's instructions. Briefly, cells subjected to different treatments were collected and processed with the kit‐provided extraction buffer. After centrifugation, the supernatants were collected for analysis. To selectively quantify NADPH, the extracts were treated following the kit protocol to remove/convert NADP^+^ and then incubated with the supplied reaction mixture. The signal was recorded using a microplate reader. NADPH levels were normalized proportionally across different treatment groups.

### 4.26 JC‐1 Staining

A375 cells (3 × 10^5^) were seeded in confocal dishes and cultured for 24 h to ensure adhesion. The culture medium was then replaced, and the cells were treated with nanoparticles from different groups for an additional 24 h. Subsequently, the cells were irradiated with L for 10 min, followed by further incubation for 6 h. The medium was then replaced with 1 mL of JC‐1 staining working solution, and the cells were incubated for 30 min. After staining, the cells were washed three times with PBS and subjected to fluorescence imaging.

### 4.27 MPTP Detection

A375 cells (3 × 10^5^) were seeded in confocal dishes and cultured for 24 h to ensure adhesion. The culture medium was then replaced, and the cells were treated with nanoparticles from different groups for an additional 24 h. After treatment, the medium was replaced with 1 mL of staining working solution containing calcein and CoCl_2_ and incubated for 30 min. The cells were subsequently stained with Hoechst 33342 and imaged by CLSM.

### 4.28 Mitochondrial ROS Detection

A375 cells (3 × 10^5^) were seeded in confocal dishes and cultured for 24 h to ensure adhesion. The culture medium was then replaced, and the cells were treated with nanoparticles from different groups for an additional 24 h. Following treatment, the medium was replaced with 1 mL of fresh medium containing MitoSOX Red Mitochondrial Superoxide Indicator (Invitrogen, Thermo Fisher Scientific, Cat. No. M36008), a mitochondria‐targeted dihydroethidium/hydroethidine derivative bearing a triphenylphosphonium moiety for live‐cell imaging, and the cells were incubated for 30 min. The cells were then stained with Hoechst 33342 and imaged by CLSM.

### 4.29 γ‐H2AX, CRT and HMGB1 Immunofluorescence Staining

A375 cells (3 × 10^5^) were seeded in confocal dishes and cultured for 24 h to ensure adhesion. After treatment, the cells were fixed with 4% paraformaldehyde (PFA). The cells were then incubated overnight at 4°C with primary antibodies against γ‐H2AX (1:100), calreticulin (CRT, 1:100), and high‐mobility group box 1 (HMGB1, 1:100). Subsequently, the cells were stained with DAPI for 5 min at room temperature and imaged by CLSM.

### 4.30 ATP Determination

Intracellular ATP levels were measured using an Enhanced ATP Assay Kit (Beyotime, Shanghai, China). Cells subjected to different nano‐formulations were collected and lysed with 200 µL of ATP lysis buffer according to the manufacturer's instructions. The lysates were centrifuged to obtain the supernatants, which were then mixed with the ATP detection reagent. The luminescence intensity was immediately measured using a chemiluminescence detector.

### 4.31 Western Blot

Following different treatments, A375 cells were lysed in the RIPA buffer. Protein concentrations were quantified, and equal amounts of protein from each group were separated by SDS–PAGE using a 10% precast gel (Bio‐Rad, Finland) at 120 V for 1 h. After electrophoresis, the proteins were transferred onto polyvinylidene fluoride membranes using a Trans‐Blot SD system (Bio‐Rad) at 300 mA for 90 min. The membranes were then blocked with 5% nonfat milk for 1 h at room temperature. The membranes were incubated overnight at 4°C with primary antibodies against glucose‐6‐phosphate dehydrogenase (G6PD, 1:500), HSP90 (1:500), cytochrome c oxidase subunit IV (COX IV, 1:500), and β‐actin (1:1000). After washing three times with Tris‐buffered saline containing 0.1% Tween‐20, the membranes were incubated with appropriate secondary antibodies (1:2000) for 1 h at room temperature. Protein bands were visualized using enhanced chemiluminescence and imaged with a ChemiDoc Imaging System (Bio‐Rad).

### 4.32 Construction of Subcutaneous A375 Melanoma Model

A subcutaneous melanoma model was established using BALB/c nude mice (SPF grade, 4–5 weeks old, 15–20 g). After disinfection of the injection site, A375 melanoma cells (1 × 10^6^ cells suspended in 100 µL of PBS) were subcutaneously injected into the flank of each mouse using an insulin syringe. Following injection, the mice were placed on a heating pad until full recovery and then housed under standard conditions (temperature 22°C–26°C, humidity 50%–60%) with free access to food and water. All animal experiments were approved by the Institutional Animal Care and Use Committee of WIUCAS (Approval No. WIUCAS25122302) and conducted in accordance with institutional guidelines.

### 4.33 In Vivo Biodistribution and Infrared Thermography

To evaluate the in vivo biodistribution of nano‐formulations, two groups, JAPC/A‐Cy5 or JAPC/A‐Cy5@CM, were used for analysis of the targeting efficiency. Briefly, nano‐formulations (30 mg/kg) were injected via the tail vein, and the mice were imaged in vivo at 12 h. The heart, liver, spleen, lung, and kidney were collected and imaged to assess the targeting effect. To further evaluate in vivo photothermal conversion performance, the mice were treated with 100 µL JAPC/A@CM and then exposed to 1 W cm^−2^ 808 nm irradiation for 5 min. The temperature change was monitored with the FLIR E76 Infrared Thermal Imager.

### 4.34 In Vivo Anticancer Treatment and Histopathology Staining

Mice bearing subcutaneous A375 melanoma tumors were randomly divided into five groups: PBS, JAPC@CM, JAPC/A@CM, JAPC@CM + L, and JAPC/A@CM + L. JAPC/A@CM indicates cell membrane–coated JAPC/A, and L indicates irradiation with an 808 nm NIR laser.

On day 1, nano‐formulations (30 mg/kg) from the respective groups were intravenously administered via the tail vein. After 12 h, mice in the laser‐treated groups were exposed to 808 nm laser irradiation at a power density of 0.5 W cm^−^^2^ for 5 min. During mild photothermal therapy, the temperature at the tumor site was continuously monitored using real‐time infrared thermographic imaging and precisely maintained at approximately 42°C. Treatments were performed every 3 days, and tumor volume was recorded every 3 days and calculated using the formula: V = (length × width^2^) / 2. On day 16, the mice were sacrificed, and major organs, including the heart, tumor tissue, liver, spleen, lung, and kidney, were collected for histopathological analysis. The harvested tissues were fixed in paraformaldehyde, dehydrated, embedded in paraffin, and sectioned into 5 µm slices. The tissue sections were treated with proteinase K at 37°C for 30 min and subsequently subjected to H&E staining, as well as immunofluorescence or immunohistochemical staining for Ki67 (1:1000), TUNEL reaction mixture (Servicebio, China), HSP90 (1:500), cytochrome c (1:500), RNS probe (Bestbio, Shanghai, China), HMGB1 (1:500), and CRT (1:500). After washing three times with PBS (pH 7.4), the sections were incubated with appropriate secondary antibodies for 60 min, cover‐slipped with neutral balsam, and imaged using an inverted fluorescence microscope (Axio Observer, Zeiss).

### 4.35 Statistics and Reproducibility

Data analysis was performed using Origin 2024b and GraphPad Prism 9.5. Statistical significance was evaluated by one‐way ANOVA with Tukey's post hoc test. All results are presented as the mean ± standard deviation. All collected samples and animals were included in the analysis, and no data points were excluded. The symbol # in the figure annotations denotes statistical significance at *p* < 0.001.

## Author Contributions

**Chengcheng Li** contributed to material design and manuscript writing and performed most of the experiments. **Yu Zhang** and **Yu Chen** contributed to parts of the cell‐based experiments. **Lingyun He** contributed to parts of the animal experiments. **Xian Shen**, **Weijian Sun**, and **Hongbo Zhang** jointly supervised, funded, and reviewed this work.

## Conflicts of Interest

The authors declare no conflicts of interest.

## Supporting information




**Supporting File**: advs77848‐sup‐0001‐SuppMat.docx.

## Data Availability

The data that support the findings of this study are available on request from the corresponding author. The data are not publicly available due to privacy or ethical restrictions.
