# Supplementary material for: Asymmetric Structure‐Driven Functional Synergy: A Multistimuli‐Activated Janus Nanomotor for Self‐Amplifying NO Cascade Therapy
Source: Adv Sci (Weinh). 2026 Sep 27:e77848. Online ahead of print. doi: 10.1002/advs.77848 (PMC13616278; doi:10.1002/advs.77848)
Supplement: Supplementary file 1 [file ADVS-9999-e77848-s001.docx]

**Supporting Information**

**Asymmetric Structure-Driven Functional Synergy: A Multistimuli-Activated Janus Nanomotor for Self-Amplifying NO Cascade Therapy**

Chengcheng Li, Yu Zhang, Yu Chen, Lingyun He, Xian Shen*, Weijian Sun*, Hongbo Zhang*

C. Li, L. He, X. Shen, W. Sun, H. Zhang

Department of Colorectal and Anal Surgery, Zhejiang–Finland Joint Laboratory of Gastrointestinal Tumor Metabolism and Nutrition, The First Affiliated Hospital of Wenzhou Medical University, Wenzhou, Zhejiang, China

E-mail: Hongbo Zhang：[hongbo.zhang@abo.fi](mailto:hongbo.zhang@abo.fi); Weijian Sun: [fame198288@126.com](mailto:fame198288@126.com); Xian Shen：[shenxian@wmu.edu.cn](mailto:shenxian@wmu.edu.cn).

C. Li, Y. Zhang, Y. Chen, H. Zhang

Pharmaceutical Sciences Laboratory, Faculty of Science and Engineering, Åbo Akademi University, Turku, Finland

H. Zhang

Turku Bioscience Centre, University of Turku and Åbo Akademi University, Turku, Finland

*Corresponding author

**Figure S1.** SEM images of JAPC.

**Figure S2.** The element line-scanning profile and EDS spectrum of JAPC.

**Figure S3.** XPS spectra of JAPC.

**Figure S4.** POD activity of JAPC/A was evaluated by the TMB assay.

**Figure S5.** Photographic images of bubbles generation from AuPt₃ and AuPt₃Cu nanozyme under the same conditions.

**Figure S6.** Photographic images of bubbles generation from various groups.

**Figure S7.** Fluorescence intensity quantification of the cellular uptake of JAPC/A and JAPC/A@CM. Data are presented as mean ± SD, *n* = 3.

**H₂O₂ + L-Arginine + NIR**

**Blank**

**H₂O₂**

**H₂O₂ + L-Arginine**

**Figure S8.** Photographic images of the trajectory of JAPC/A under various physicochemical stimuli conditions.

**Figure S9.** Quantitative analysis of intracellular GSH levels. Data are presented as mean ± SD, *n* = 3. ^#^*p* < 0.001 versus the control group.

**Figure S10.** Quantitative analysis of intracellular NO levels. Data are presented as mean ± SD, *n* = 3. ^#^*p* < 0.001 versus the control group.

**Figure S11.** Photographic images of the mouse under inhalation anesthesia during photothermal imaging.

**Figure S12.** The body weights of mice are from different groups. Data are presented as mean ± SD, *n* = 4.

**Figure S13.** Representative H&E-stained histological images of major organs from different treatment groups. Scale bars: 50 μm.

**Figure S14.** Representative images of RNS staining from different treatment groups. Scale bars: 50 μm.

**Figure S15.** Representative images of HSP90 staining from different treatment groups. Scale bars: 50 μm.

**Figure S16.** Representative images of HMGB1 and CRT staining from different treatment groups. Scale bars: 20 μm.

**Figure S17.** Representative images of CD80 (green) and CD86 (red) staining from different treatment groups. Scale bars: 20 μm.
